# Functionalized Hydrogel-Based Wearable Gas and Humidity Sensors

**DOI:** 10.1007/s40820-023-01109-2

**Published:** 2023-05-24

**Authors:** Yibing Luo, Jianye Li, Qiongling Ding, Hao Wang, Chuan Liu, Jin Wu

**Affiliations:** State Key Laboratory of Optoelectronic Materials and Technologies and the Guangdong Province Key Laboratory of Display Material and Technology, School of Electronics and Information Technology, Sun Yat-Sen University, Guangzhou, 510275 People’s Republic of China

**Keywords:** Health and safety monitoring, Gas and humidity sensor, Functionalized hydrogel, Wearable sensor, Flexible and stretchable sensor

## Abstract

Breathing is an inherent human activity; however, the composition of the air we inhale and gas exhale remains unknown to us. To address this, wearable vapor sensors can help people monitor air composition in real time to avoid underlying risks, and for the early detection and treatment of diseases for home healthcare. Hydrogels with three-dimensional polymer networks and large amounts of water molecules are naturally flexible and stretchable. Functionalized hydrogels are intrinsically conductive, self-healing, self-adhesive, biocompatible, and room-temperature sensitive. Compared with traditional rigid vapor sensors, hydrogel-based gas and humidity sensors can directly fit human skin or clothing, and are more suitable for real-time monitoring of personal health and safety. In this review, current studies on hydrogel-based vapor sensors are investigated. The required properties and optimization methods of wearable hydrogel-based sensors are introduced. Subsequently, existing reports on the response mechanisms of hydrogel-based gas and humidity sensors are summarized. Related works on hydrogel-based vapor sensors for their application in personal health and safety monitoring are presented. Moreover, the potential of hydrogels in the field of vapor sensing is elucidated. Finally, the current research status, challenges, and future trends of hydrogel gas/humidity sensing are discussed.

## Highlights

- A systematic summary of the research progress of hydrogel-based gas and humidity sensors is presented.
- The sensing mechanism of hydrogel-based gas and humidity sensors is elaborated.
- The potential of hydrogel-based vapor sensors in different fields of application is demonstrated.

## Graphical abstract

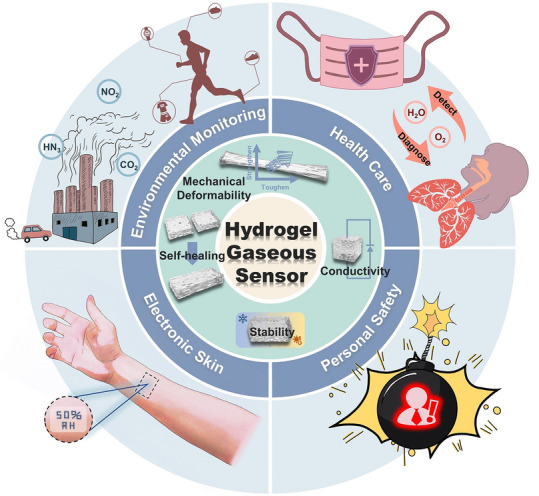

## Introduction

With the rapid growth of industrialization and urbanization, the quality of life has taken a notable leap; however, this consequently increased environmental problems. Polluting gases, such as NO_2_, NH_3_, CO, and H_2_S, and volatile organic compounds, including benzene, ethanol, and formaldehyde, are emitted into the environment by vehicle exhaust and factories. When their concentration exceeds a certain threshold, which is as low as 1 ppm, this causes indelible harm to human health and the environment [1–11]. Moreover, flammable and explosive gases, such as H_2_ and CH_4_, pose a direct threat to the safety of human life and property [12–17]. To avoid underlying risks, we require devices that can accurately detect the presence and concentration of these toxic, flammable, and explosive gases in real time; thereby, prompting appropriate protective measures.

Vapor-sensing devices are often found in the medical and health care industries [18–20]. As human exhalation contains a large number of gas and water molecules, monitoring and analyzing information related to it, such as respiratory rate and composition of exhalation, can achieve the non-invasive and comfortable early prevention and treatment of certain diseases [21, 22]. For example, the acetone concentration in the breath can be used as a biomarker for diabetes, and the detection of breath humidity enables respiratory monitoring [23–26]. However, expensive and bulky testing equipment and complex operational requirements, which require testing at a specific location guided by professionals only, limit the accessibility of the technology to ordinary people. Therefore, portable and wearable gas/humidity sensors are better alternatives for the easy and real-time monitoring of health and safety, compared to bulky and expensive large instruments, such as gas chromatographs. In addition, by setting certain alarm thresholds, people can detect toxic, flammable, and explosive gases in their environment to avoid unnecessary danger or detect certain diseases at the early stage to avoid their progression.

For portable and wearable sensors, flexible and wearable electronic systems have received copious attention because of their huge potential applications in human–machine interfaces, health monitoring, and smart skins for robots [27–36]. Flexible and wearable vapor sensors can fit directly on human skin or clothing, and endure human movement without mechanical damage, which greatly broaden their application [37–39]. Moreover, they can achieve real time monitoring of target data, thereby allowing people to determine their surrounding environment and physiological health status anytime and anywhere. In contrast, traditional gas/humidity sensors, such as semiconductor, electrochemical, and optical gas sensors, have limited mechanical deformability, which makes them inherently unsuitable for flexible and wearable devices. For flexible applications, sensitive materials are commonly integrated on a flexible substrate [40–43]. However, devices prepared using this approach have limited strain tolerance (typically ≤ 50%) and poor adaptation of materials with different mechanical properties [44]. Moreover, based on the application of wearable devices, sensors are expected to operate at room temperature, which reduces additional heating power consumption and decreases thermal risks, such as burns. For example, widely studied metal–oxide semiconductor vapor sensors require high temperatures to ensure the sensing operation of their sensitive material, which is contrary to the operation of wearable devices [45–47]. Therefore, the combined requirements for wearable gas/humidity sensors, such as stretchability and room-temperature operation, prompt the need for a novel sensitive material that can achieve high strength, stretchability, and room-temperature sensibility.

Hydrogel is a gel composed of polymer network chains and a large amount of water molecules with a three-dimensional network structure [48–50]. It is extremely hydrophilic and rapidly swells in water to retain a large volume of water without dissolving [51, 52]. The hydrophilic functional groups on the polymer backbone endows the water absorption of hydrogels, whereas the crosslinking between the polymer network chains resist dissolution [53]. Thus, hydrogels are permeable to chemical and biological molecules and transparent to light and sound waves, which are liquid-like properties that are attributed to their high-water content. Meanwhile, the crosslinked structure of the polymer network chains endows the hydrogels with flexibility and stretchability, similar to that of elastic solids [52].

Since 1954, when Wichterle and Lim first synthesized hydrogel [54], it has been widely used in various fields, including food preparation, bioengineering, agriculture, healthcare, and biosensors [55–63]. For different application requirements, hydrogels can be functionalized to achieve specific properties and structures [64–66]. Thus, functionalized hydrogels are more suited to the complex requirements for flexible and wearable vapor sensors. In particular, it allows gas and humidity sensing at room temperature, thereby reducing the risk of explosion, while reducing the power consumption. In addition, its deformability allows the sensor to fit more closely to complex curved surfaces, such as human skin, and follow various body movements without mechanical damage. The intrinsic conductivity allows hydrogel-based vapor sensors to directly respond to electrical parameters for easier collection and processing. Self-healing ability permits the sensor to heal without changing its response after mechanical damage, thereby extending its lifespan. High transparency and biocompatibility enhance the aesthetic wearability and potential of hydrogel-based vapor sensors for medical applications. Recent studies have reported the use of hydrogels as the sensitive material for gas-sensing applications, such as NO_2_, NH_3_, CO_2_, and O_2_ [67–75]. In addition, hydrogels have a unique swelling property that makes them naturally sensitive to humidity for humidity sensing. Several studies reported the direct use of hydrogel-based vapor sensors for practical applications, such as smart masks, electronic skin, respiratory analysis, and wireless monitoring, demonstrating their great prospects for applications in environmental monitoring, medical health, and other fields [76–79]. In contrast to flexible sensing schemes that integrate sensitive materials in an elastic substrate, the intrinsically sensitive, conductive, and stretchable properties of hydrogels have increased the efficiency and convenience for the preparation of sensors. By attaching hydrogel vapor sensors to the human skin or integrating them into clothing and accessories, personal health and safety monitoring can be easily achieved without additional equipment.

**Fig. 1:**
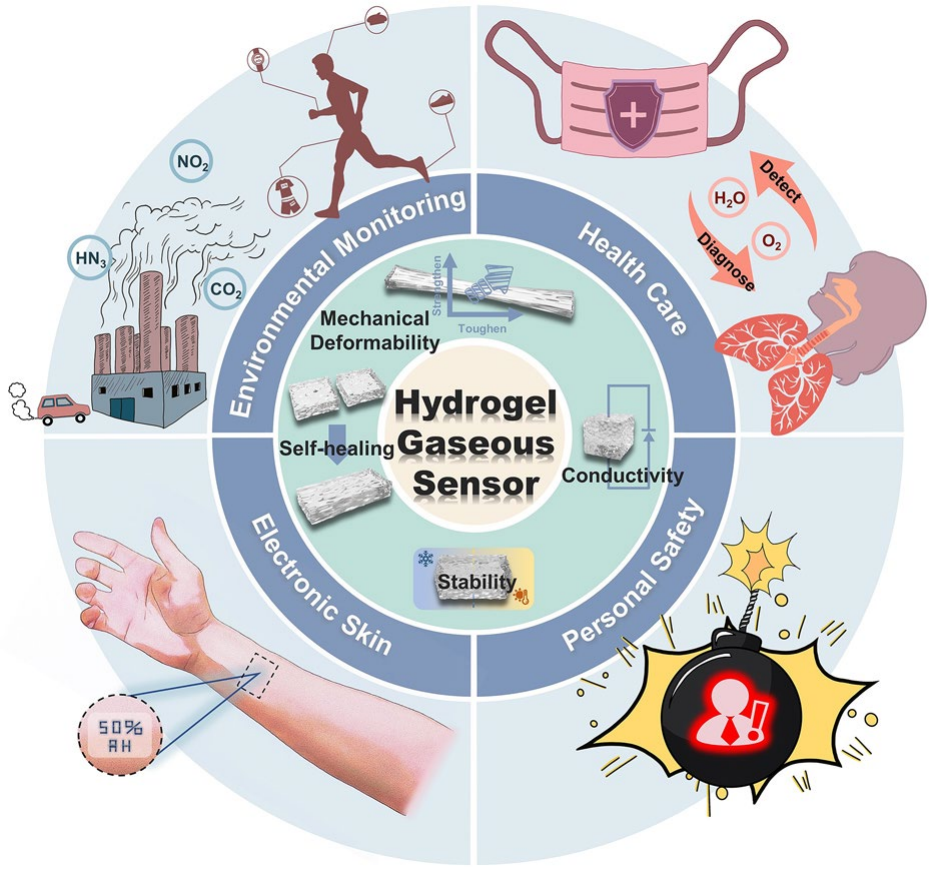
Schematic of the functionalized hydrogel-based vapor sensors for personal health and safety monitoring

Breathing is an inherent human activity; however, the composition of the air we inhale and gas we exhale remains unknown. Using hydrogel as the sensitive material, flexible stretchable and wearable vapor sensors can acquire the information of the gas content and concentration in the inhaled air and exhaled gas through the continuous testing of ambient air or human exhaled gas at room temperature, thereby realizing the real-time monitoring of personal health and safety. Currently, reviews have focused on hydrogel research in vapor sensing when introducing recent work on flexible wearable sensors, or describing research on hydrogels [80–84]. However, only general information on hydrogel-based vapor sensors is provided, and a systematic overview of hydrogel research in gas/humidity sensing and their applications for personal health and safety monitoring is not available. The research on hydrogel-based gas/humidity sensors is increasing, and concerns about health and safety are growing. Therefore, a review focused on the research of hydrogel-based gas/humidity sensors and their role on personal health and safety is needed. This paper presents a well-rounded discussion of hydrogel-based vapor sensors in terms of their properties and optimization, existing mechanisms and applications for personal health and safety monitoring (Fig. 1). The current research status and problems to be solved for hydrogel-based vapor sensors are summarized. Finally, the conceivable future research trends for hydrogel gas/humidity sensing are discussed.

## Design and Optimization of Hydrogels

Hydrogels are classified based on their properties. According to the source, hydrogels can be divided into natural and synthetic hydrogels [85–87]. Natural hydrogels, such as collagen, chitosan (CS), hyaluronic acid, alginate, gelatin, elastin, chondroitin sulfate, and heparin, have excellent biocompatibility, and are used in a wide range of applications, such as bioengineering, medical health, and food processing [88–94]. Nonetheless, their unsatisfactory mechanical deformability, uncontrollable structure, and degradation limit their broader spectrum of applications [48, 49]. Meanwhile, synthetic hydrogels with enhanced mechanical deformability and controlled structure and degradation, such as polyethylene glycol (PEG), polyvinyl alcohol (PVA), poly(2-hydroxyethyl methacrylate) (PHEMA) and polyacrylamide (PAM), can be used in sensors, actuators, soft robots, and wastewater treatment [95–101]. However, synthetic hydrogels have lower biocompatibility than that of natural hydrogels, making them unsuited for health-related applications [48, 49]. According to the different methods of crosslinking polymer chains, they can be divided into physically and chemically crosslinked hydrogels [85–87]. Physically crosslinked hydrogels are formed by various strong/weak intermolecular interactions between polymer chains and usually have poor mechanical deformability, which limit their applications [102–104]. In contrast, chemically crosslinked hydrogels are crosslinked by covalent bonding between polymer chains, resulting in superior mechanical deformability [105–107]. However, the degradation of biocompatibility owing to the residual chemical crosslinkers, organic solvents, and photoinitiators in chemically crosslinked hydrogels limit their usage in biomedical-related applications.

Based on the practical application of wearable vapor sensors, large deformability, long-term stability, conductivity, self-healing, high responsiveness, and biocompatibility are desired. As different hydrogels have their own advantages and disadvantages, simple hydrogels cannot meet these complex requirements for stretchable and wearable vapor sensors. Hence, simple hydrogels should be optimized and modified to meet practical application demands. In the recent decades, important advances have focused on the preparation of hydrogels with the modulation of their properties, such as porosity, biocompatibility, electrical conductivity, and mechanical deformability, by different design and optimization approaches to meet different application requirements. In this section, we will briefly introduce some inherent and functionalized properties of hydrogels, and the corresponding design and optimization approaches from the application requirements of flexible wearable vapor sensors to provide possible theoretical support for the design and preparation of gas/humidity sensors for personal health and safety monitoring.

### Mechanical Deformability

Flexibility and stretchability are important requirements for hydrogel-based wearable vapor sensors. These properties enable the sensor to tightly fit to irregular surfaces, such as human skin, thereby ensuring the user’s comfort while wearing the sensor. In addition, the sensor can stretch without being damaged with body movement. During usage, various degrees of mechanical deformation or even mechanical damage are generated in the actual operation. This requires the flexibility and robust mechanical properties of the sensor, while maintaining its high sensitivity. However, conventional hydrogels have low mechanical deformability with a small fracture energy, low elastic modulus, and brittleness, which does not meet the large mechanical deformability demand for flexible and wearable vapor sensors [108–110]. Thus, various technical approaches need to be introduced to enhance the toughness and stretchability of hydrogels for practical applications.

**Fig. 2:**
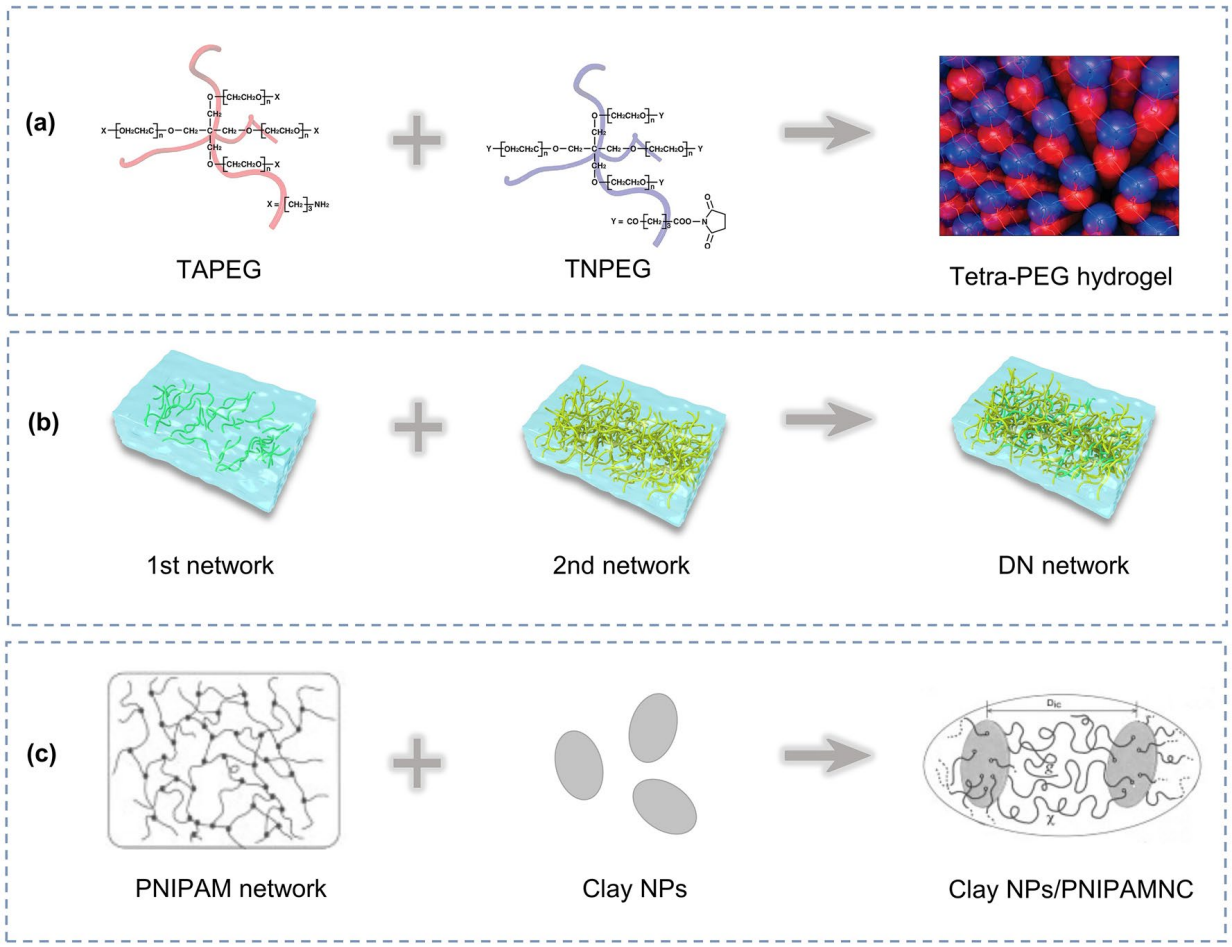
**a** Schematic of the synthesis of tetra-PEG hydrogel. Reproduced with permission [111]. Copyright 2008, American Chemical Society. **b** Schematic of the synthesis of DN hydrogels. **c** Schematic diagram of the synthesis of clay NPs/PNIPAMNC. Reproduced with permission [112]. Copyright 2002, Wiley‐VCH

The direct crosslinking of polymer chains or free polymerization of vinyl monomers with crosslinkers is the conventional approach to prepare hydrogels. Nonetheless, the reflective activity differences between the species leads to the fracture of the hydrogel owing to the local stress concentration under external forces. The uneven crosslinking density between polymer chains, varying chain lengths between crosslinking points, and presence of dangling chains and rings are the factors that contribute to the localized stress concentration [113–115]. Therefore, increasing the homogeneity of hydrogel and avoiding local stress concentration can improve the mechanical deformability of hydrogels. Sakai et al. designed a homogeneous tetra-polyethylene glycol hydrogel [111]. They synthesized symmetric tetrahedral macromolecular monomer phases with the same PEG arm length, as shown in Fig. 2a. The two monomers were terminal tetraamine polyethylene glycol (TAPEG) and terminal tetra-NHS polyethylene glycol (TNPEG). By adjusting the stoichiometric ratio of the two monomers, a series of hydrogels is prepared. The hydrogels prepared with a stoichiometric ratio of 1:1 had a maximum compressive strength of 2.5 MPa, which was five times higher than that of conventional agarose or acrylamide gels. In their subsequent studies, the importance of the homogeneity of polymer crosslinking in enhancing the mechanical deformability of hydrogels was demonstrated [116, 117].

The mechanical deformability of hydrogels can also be enhanced by introducing mechanical dissipation mechanisms. Several microcracks and nanocracks are inevitably introduced during the preparation and processing of hydrogels. These microcracks and nanocracks can easily be propagated under stress application, resulting in fracture of the hydrogel at the macroscopic level. With the introduction of a mechanical dissipation mechanism, the cracks in hydrogels are inhibited. A double-network (DN) structure is a common approach for introducing a mechanical energy dissipation mechanism. The dual network is composed of polymer network chains with opposite properties [104, 118–122]. One polymer network is composed of a soft, ductile, and loosely crosslinked polymer with high molecular weight, whereas the other polymer network is composed of a rigid, brittle, and densely crosslinked polymer, as illustrated in Fig. 2b. During the stretching of DN hydrogels, the mechanical energy is dissipated by sacrificing the breakage of chemical bonds in the densely crosslinked polymer network, or by the perturbation of non-covalent interactions, thereby improving the mechanical deformability of the hydrogel [123–125]. Wu et al. [126] used a one-pot method to prepare PAM/Carrageenan (Carr) DN hydrogels for NO_2_ and NH_3_ sensing. The DN hydrogel has an elastic modulus of 299 kPa, which is four times higher than that of a single-network Carr hydrogel and 33 times higher than that of a single-network PAM hydrogel. This implies that the deformability of DN PAN/Carr hydrogels is significantly improved and can be adapted to the needs of flexible wearable vapor sensor applications.

Combining the two toughening mechanisms mentioned above is a good approach to improve the mechanical deformability of hydrogels. Haraguchi et al. proposed nanocomposite (NC) hydrogels [112], as shown in Fig. 2c. They prepared clay nanoparticles (NPs)/poly(N-isopropyl acrylamide) NC (PNIPAMNC) hydrogels using a NIPAMNC monomer and well-dispersed clay NPs. Multiple polymer chains are linked to a single clay NP under various non-covalent interactions, such as electrostatic and coordination interactions. The homogeneous dispersion of the clay NPs in the flexible network alleviated the problem of localized stress concentration, whereas the non-covalent interactions between the clay NPs and polymer chains interfere with the dissipation of mechanical energy. By the superposition of the two toughening effects, the hydrogel could reach a tensile strength of 109 kPa and elongation of 1.424%.

### Conductivity

Conductivity is an important characteristic of hydrogel-based vapor sensors. The change in electrical parameters as the response signal makes the subsequent signal collection and processing easier and more convenient. Thus, the conductivity of the hydrogel has notable effects on its gas/humidity response performance. In particular, modulating the conductivity of the hydrogel according to the actual usage requirements can effectively improve the response characteristics of the vapor sensor. In addition, hydrogels with intrinsic conductivity have high flexibility, stretchability, and biocompatibility, which allow them to meet certain special application scenarios. The inherent stretchability eliminates the need for hydrogels to integrate additional substrates for the fabrication of sensors, thereby avoiding a series of complex mechanical engineering and laminating processes, and unsuitable fit after lamination. However, traditional hydrogels are not inherently conductive. Thus, different special hydrogel treatments are required to modulate this property.

**Fig. 3:**
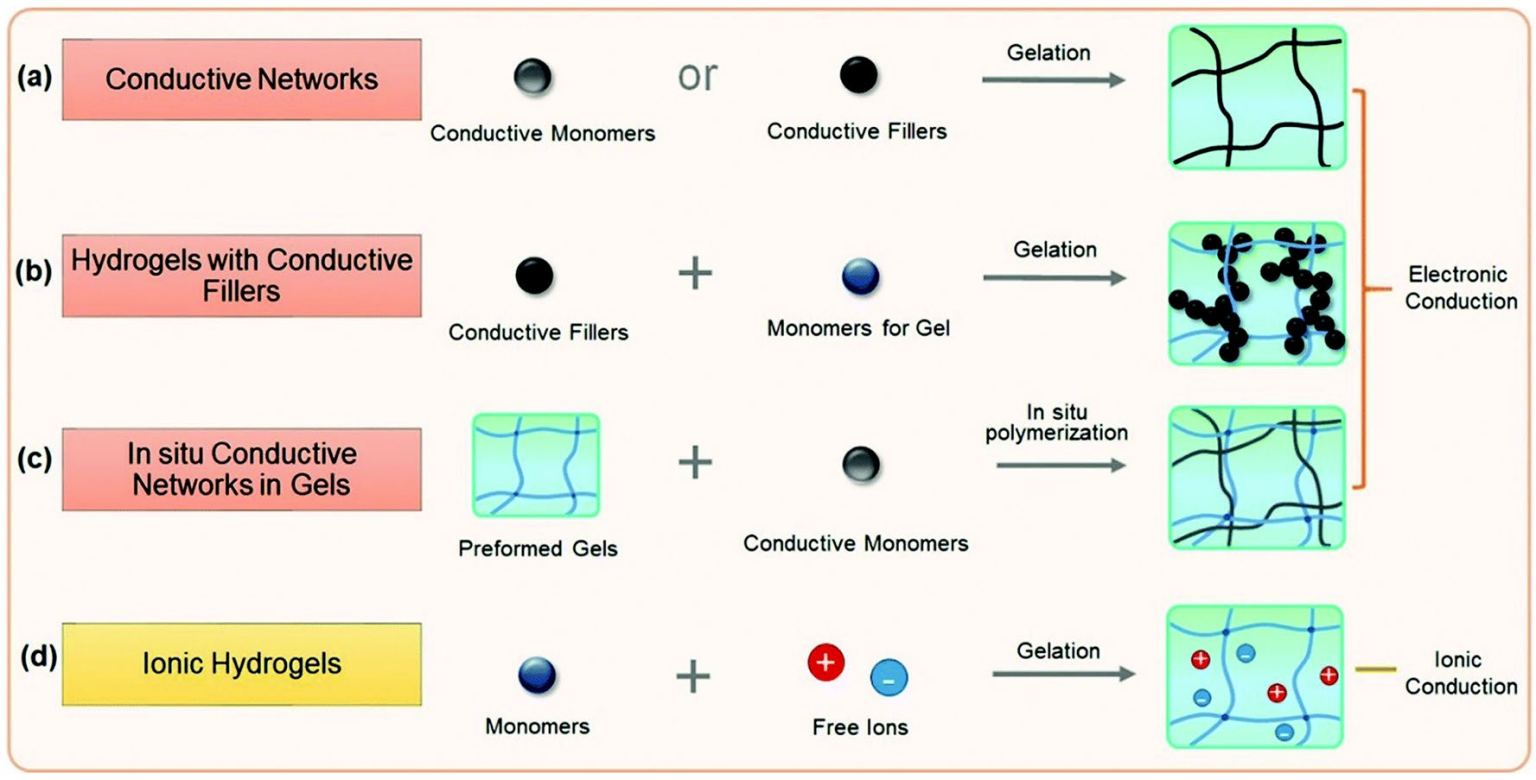
Main approaches for producing conductive hydrogels. Reproduced with permission [127]. Copyright 2020, Royal Society of Chemistry

As shown in Fig. 3, conductive hydrogels can be divided into electron- and ion-conductive hydrogels, according to their conductive modes. Direct crosslinking of conductive materials is used to synthesize electrically conductive hydrogels, as shown in Fig. 3a. Common conductive materials such as graphene, carbon nanotubes, and conductive polymers like polyaniline (PANI), polypyrrole (PPY), and poly(3,4-ethylenedioxythiophene) (PEDOT) are commercially available [128–134]. They can be directly crosslinked by doping molecules to form conductive hydrogels. Reduced graphene oxide hydrogel (RGOH) is synthesized from graphene oxide using a one-pot hydrothermal self-assembly method by Wu et al. [135]. This hydrogel is electronically conductive, and can be used for NO_2_ and NH_3_ sensing. Another common method is polymerization and gelation of the hydrogel precursor solution with conductive fillers, such as metal NPs, carbon nanotubes, and conductive polymers, as depicted in Fig. 3b [136–140]. In addition, electron-conductive hydrogels can be prepared using a non-conductive hydrogel as the substrate carrying a conductive polymer precursor, as depicted in Fig. 3c, in which the conductive polymer polymerizes in situ to form an interpenetrating network conductive hydrogel with uniform density [141–145]. In existing vapor sensor research, ion-conductive hydrogels are more commonly used as sensing materials. This type of hydrogels achieves their conductive properties with the addition of salt solution with high concentration, thereby introducing a large number of free ions (Fig. 3d) [127, 146, 147]. The ionic conductivity of hydrogels is commonly enhanced by preparing polyelectrolyte networks or constructing ion channels in hydrogel networks [148, 149]. Wu et al. [150] used the salt infiltration method to obtain ion-conductive hydrogels by adding calcium chloride (CaCl_2_) to PAM/Carr DN hydrogels. The addition of CaCl_2_ enhanced the conductivity, inhibited the dissolution of NH_3_ and other gases, and improved the NO_2_ selectivity of the hydrogel. In addition, it promoted the redox NO_2_ reaction in the hydrogel and electrode, which enhanced the response characteristics of the hydrogel.

### Anti-Freezing and Water Retention Abilities

The operation of wearable vapor sensors follows the work and living environment of the wearer; thus, they are likely to be subjected to complex environments, such as sub-zero temperatures, or harsh or dry environments. However, traditional hydrogel materials do not have this capability. At low temperatures, the water molecules in the hydrogel tend to condense into ice, which eventually leads to freezing, stiffness, brittleness, and loss of working ability of the hydrogel. In low-humidity or high-temperature environments, the water molecules in the hydrogel easily volatilize and evaporate, resulting in the excessive water loss in the hydrogel, making them easy to crumple, dry, harden, and lose its working ability. Therefore, to achieve hydrogel-based vapor sensor with proper operation for different complicated environments, its resistance to freezing and dehydration is an important parameter.

**Fig. 4:**
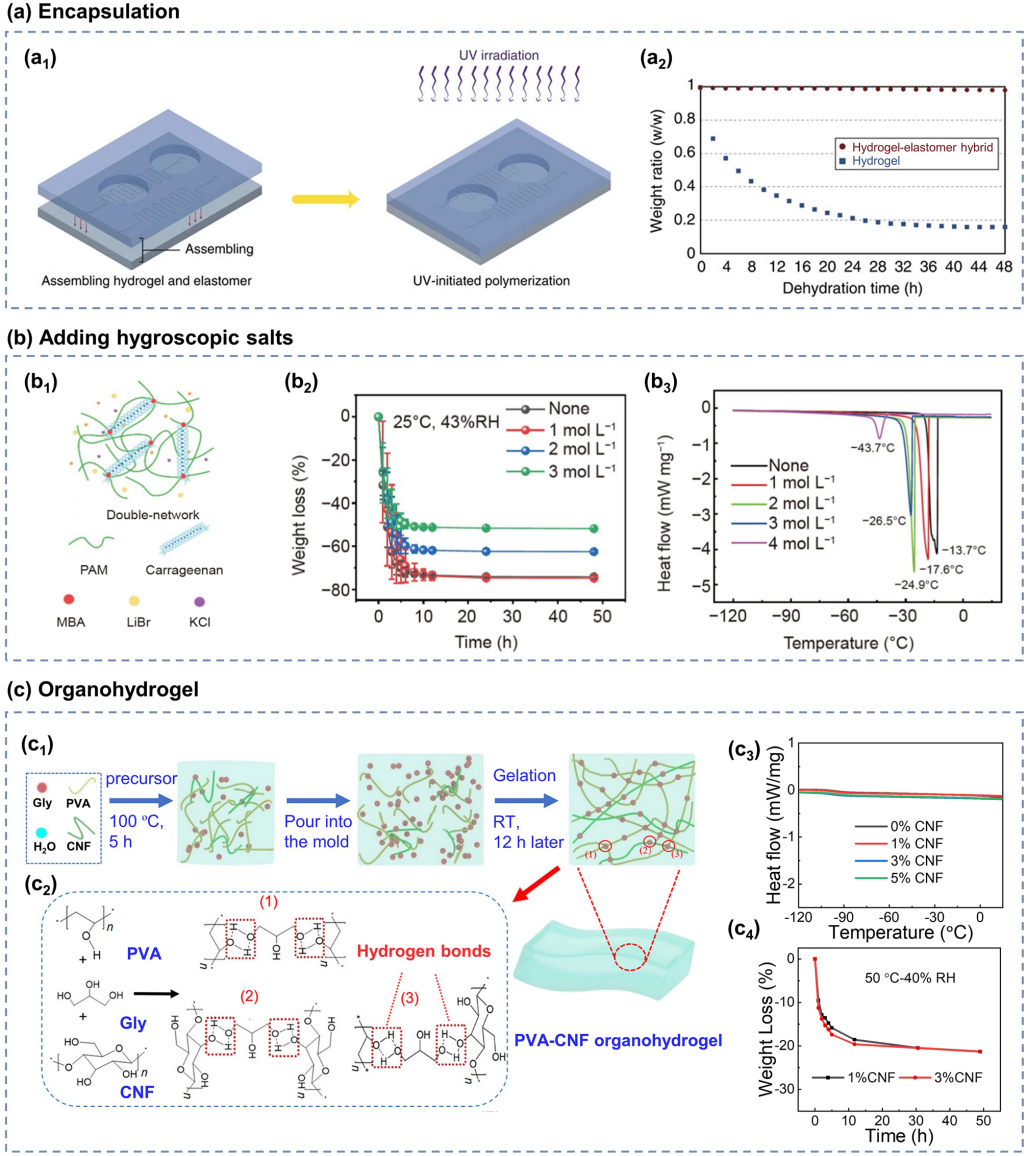
**a** Encapulation: **a**_**1**_ Schematic diagram of elastomer encapsulated hydrogel; **a**_**2**_ Variation of the weight ratio of hydrogel and hydrogel-elastomer hybrid with time. Reproduced with permission [151]. Copyright 2016, Springer Nature. **b** Adding hygroscopic salts: **b**_**1**_ Schematic structure of LiBr-PAM/Carr DN hydrogel; **b**_**2**_ Trends in weight of hydrogels with different concentrations of LiBr content over time under room temperature drying environment; **b**_**3**_ DSC spectra of these hydrogels. Reproduced with permission [152]. Copyright 2022, Springer Nature. **c** Organohydrogel: **c**_**1**_**-c**_**2**_ Schematic diagram of PVA/CNF-Gly preparation method; **c**_**3**_ DSC spectra of the organohydrogels with different CNF contents; **c**_**4**_ Weight trends over time for organic hydrogels with different CNF contents under high temperature and dry conditions. Reproduced with permission [67]. Copyright 2022, Wiley‐VCH

The encapsulation of hydrogel sensors with elastomeric materials is an effective approach to prevent the evaporation of water from hydrogels [151, 153, 154]. Yuk et al. [151] have prepared a hydrogel–elastomer hybrid with an extremely strong interfacial and functional microstructure, inspired by the structure and function of mammalian skin, as shown in Fig. 4a. The hybrid retains stretchability, while enhancing the water-retention capacity of the hydrogel, thereby enabling it to work in dry environments. Despite the improvement of the water-retention capacity by isolating the hydrogel from the external environment with elastomer encapsulation, this method has minimal effects in lowering the freezing point of the hydrogel to improve its freezing resistance. Moreover, the additional elastomer encapsulation limits the application of hydrogels for humidity and gas sensing. Another common method to enhance the freezing resistance and water storage capacity of hydrogels is the addition of hygroscopic salts, such as sodium chloride and CaCl_2_ [155–158]. Wu et al. [152] added lithium bromide (LiBr) to PAM/Carr hydrogels using the immersion method, which significantly inhibited dehydration and freezing (Fig. 4b). The PAM/Carr hydrogels without and with LiBr were simultaneously placed at a temperature of 25 °C and relative humidity (RH) of 43%. As illustrated in Fig. 4b_2_, the volume of the hydrogel without LiBr decreased significantly and became fragile after 48 h, whereas the hydrogel with LiBr has minimal variations with enhanced water storage capacity. In addition, the freezing point of the hydrogel decreased from − 13.7 to − 43.7 °C after the addition of LiBr, as shown in Fig. 4b_3_, which indicates the improved freezing resistance. In addition, the organohydrogel can be obtained by replacing a portion of the aqueous solution in the hydrogel with an organic solution to improve the defects of conventional hydrogels, such as cold and dryness resistance [159–163]. Meanwhile, this method can effectively improve the mechanical stretchability and multi-stimuli sensitivity of hydrogels. Ding et al. [67] used a facile one-pot polymerization method to prepare PVA/cellulose nanofibril (CNF)–glycerol (Gly) DN organohydrogel with significantly improved water storage and freezing resistance (Fig. 4c), which could carry out normally after placed at − 20 °C for a long time.

### Self-healing Ability

Self-healing capability refers to the ability of a material to repair and restore its original properties after being damaged. Although we can improve the mechanical deformability of hydrogel through various toughening methods for wearable vapor sensors, the devices still exhibit mechanical damage owing to various factors. If a sensor has the self-healing ability to recover and restore its original operation even after being mechanically damaged, it can extend its lifespan, and improve its reliability and durability.

**Fig. 5:**
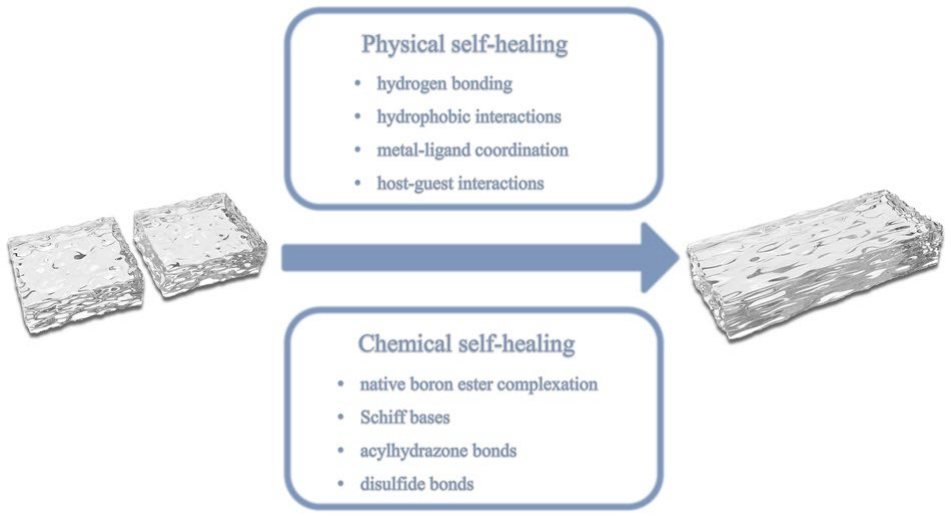
Schematic of self-healing mechanisms

The self-healing properties of hydrogels can be classified into physical and chemical self-healing, according to different mechanisms. Physical self-healing is achieved by the polymer chain reconfiguration under reversible non-covalent interactions, such as hydrogen bonding, hydrophobic interactions, metal–ligand coordination, host–guest interactions, and combinations of multiple intermolecular interactions [164–171]. In contrast, chemical self-healing is achieved by the dynamic covalent chemistry in hydrogels, including native boron ester complexation, Schiff bases, acylhydrazone bonds, disulfide bonds, and other dynamic chemical bonds and reactions [172–177]. The general reversible interactions in physical and chemical self-healing are shown in Fig. 5.

The self-healing properties of hydrogels can be achieved by designing specific molecular structures. For example, introducing more hydrogen bonding donor/acceptor groups, such as –OH, –NH_2_, and –COOH, can increase the self-healing ability of hydrogels by reversibly forming hydrogen bonds when the hydrogel is mechanically damaged. Zhang et al. [178] used the freezing/thawing method to prepare a PVA hydrogels with self-healing ability against mechanical damage at room-temperature. This self-healing process can occur without additional stimuli or healing agents. Instead, self-healing occurred when the cutting surface possesses a sufficient amount of free hydroxyl groups and a sufficiently high rate of chain migration to the cutting surface. Hence, adding more hydrogen bonding donor/acceptor groups, such as hydroxyl groups, can promote the self-healing of hydrogels. In addition, self-healing can be achieved by the hydrophobic part of hydrogels by the autonomous loading between hydrophobic structural domains/conjugation, metal–ligand coordination by the reversible chelation between ligands and metal atoms, and host–guest by combining interactions with different binding affinities [179–184]. Therefore, these relevant functional groups can be introduced into the hydrogel to realize their physical self-healing. Furthermore, these interactions can be combined to form multiple intermolecular interactions [185–187]. Li et al. [188] synthesized a self-healing ABA triblock copolymer hydrogel. The reconstruction of the catechol-mediated hydrogen bonding and aromatic interactions allow the hydrogel to withstand high strain, repeatability, and rapid nondestructive self-healing.

Deng et al. [189] prepared a covalent dynamic gel using bis-acylhydrazine functionalized poly(ethylene oxide) (A_2_) and tris[(4-formylphenoxy)methyl]ethane (B_3_) as raw materials. This hydrogel formed amide bonds through the dynamic chemical reactions between aldehyde and hydrazine groups to achieve self-healing under microacidic conditions, resulting in chemical self-healing, of which the acylhydrazone bond is a common reversible interaction. However, the acylhydrazone bond can only undergo dynamic reversible reactions under microacidic conditions; thus, its applicable pH range can be broadened by introducing other dynamic covalent bonds. For example, self-healing hydrogels can be achieved by introducing corresponding functional groups, such as phenylboronic acid and diol, which can undergo a dynamic chemical reaction to generate a phenylboronic ester complexation; thiol/disulfide bond exchange reaction that form a dynamic covalent bond disulfide bond; and the amine bond Schiff base formed by the nucleophilic attack of the amine on the aldehyde group as a dynamic covalent bond [190–197]. By utilizing the dynamic reaction of aminated gelatin, adipic acid dihydrazine, and oxidized dextrose, Chen et al. [173] synthesized a self-healing hydrogel. The damaged hydrogel can recover its original properties under the dynamic action of imine and acylhydrazone bonds. Moreover, dynamic reversible reactions, such as the Diels–Alder (DA) and reversible radical reactions, can achieve chemical self-healing [198].

### Other Properties

In addition to the properties mentioned in Sects. 2.1–2.4, other properties, such as biocompatibility, biodegradation, self-adhesiveness, and transparency, broaden the application of wearable hydrogel-based vapor sensors. Hydrogels with biocompatibility are suitable for biological and medical applications because they are non-toxic and non-damaging to human tissues. During the use of the sensors, the health and safety of the human body are ensured to accomplish special requirements. Zhou et al. [199] synthesized a biocompatible hydrogel using extracellular matrix-derived biopolymer gelatin and chondroitin sulfate. The hydrogel also has self-healing and adhesive properties that can be used to seal or reattach ruptured tissues in surgical procedures, such as by injection. Liang et al. [77] used PAM and CS to prepare a PAM–CS DN hydrogel-based O_2_ sensor. This hydrogel-based O_2_ sensor has promising application on wounds for real-time monitoring of O_2_ concentration and promoted wound healing owing to the excellent biocompatibility, biodegradability, and antimicrobial properties of CS, and hydrophilic and bioinert nature of PAM.

Biodegradability is a desirable property for the specific applications of sensors, in response to the trend of green development. Hydrogels with degradability can degrade spontaneously or on demand after completing a response task. Gao et al. [200] demonstrated the successful synthesis of supramolecular polymer-reinforced hydrogels with biodegradable properties using poly(N-acryloyl 2-glycine) (PACG) and gelatin methacrylate (Gelma) as raw materials by photoinitiated polymerization. The hydrogel has a high tensile strength (1.1 MPa), high compressive strength (12.4 MPa), large Young’s modulus (320 kPa), and high compressive modulus (837 kPa). The biodegradability of the hydrogels can be regulated by adjusting the PACG/Gelma ratio. Using black phosphorus (BP) nanosheets and thermosensitive hydrogel [poly(_D_,_L_-lactide)-poly(ethylene glycol)-poly(_D_,_L_-lactide) (PDLLA–PEG–PDLLA:PLEL)] as raw materials, Shao et al. [201] prepared a novel photothermal therapy (PTT) system for the postoperative treatment of tumors. BP@PLEL hydrogel has good biodegradability and ex-vivo biocompatibility, and can gelate rapidly into a film under near-infrared irradiation, which suitable for the PTT removal of tumor tissues after tumor resection.

Self-adhesion is another important property for wearable hydrogel vapor sensors. This enhances the adhesion of the sensor to target substrates, such as clothing and skin without using additional adhesives. Strong adhesion allows the sensor to fit more closely to substrates, such as skin, to better collect target stimuli. The strong adhesion of hydrogels requires the interaction of chemistry, connection topology, and dissipative mechanics. Liang et al. [202] synthesized a series of hydrogels with good adhesion properties. They achieved self-healing as well as on-demand solubility of the hydrogels using biodynamic bonding cross-linking of ferric iron, protocatechaldehyde containing catechol and aldehyde groups, and quaternized CS (QCS). In addition, the hydrogel is injectable and biocompatible with antibacterial and hemostatic characteristics, which enable its application in treating skin incisions and wounds. Inspired by plants, Gan et al. [203] developed a plant-derived adhesive hydrogel. The hydrogel was constructed with Ag–lignin NPs, pectin, and acrylic acid as the raw materials for a dynamic catechol redox system based on the dynamic redox reaction of catechol triggered by lignin NPs. The hydrogels demonstrated long-term reproducible adhesion owing to the constant generation of catechol moieties in the redox system, high toughness, and antimicrobial properties, which highlight their applicability in surgical or other biomedical applications.

High transparency allows the invisibility of hydrogel devices for its use in optoelectronic devices, commercial electronics, and military equipment. In addition, transparent sensors are more comfortable and aesthetically pleasing for people to wear on a daily basis. Therefore, transparency is another important characteristic of wearable hydrogel-based vapor sensors. Han et al. [204] synthesized transparent, stretchable, adhesive, and conductive hydrogels by the in-situ polymerization of polydopamine-doped PPy nanofibers in a PAM network. The hydrogel can achieve a transmittance of more than 70% of the visible spectrum even at a thickness of more than 1 mm, and can meet the requirements of various bioelectronic devices, especially human–machine interfaces. With acrylamide and PVA as the primary materials, and using N,N-methylenebisacrylamide (MBAA) crosslinking, Ge et al. [205] prepared a DN conductive hydrogel with high stretchability (> 500%), high transparency (> 90%), good adhesion, and biocompatibility. The high sensitivity of pressure of 0.05 kPa^−1^ under 0–3.27 kPa enables its successful application in the detection of speech signals, airflow, and limb movements.

## Hydrogel-based Gas Sensors

In daily work and life, people are constantly exposed to unknown gases and can even step into dangerous environments with irreversible consequences if they are not careful. To address this, portable and wearable gas sensors are used to act as a second sense to accurately identify dangerous gases that cannot be easily distinguished, detect risks, and avoid dangerous areas, thereby avoiding damage to human health and safety. In addition, wearable gas sensor can be used in the medical field to achieve non-contact diagnosis and treatment of various diseases, such as diabetes and asthma.

Currently, gas sensing, including NH_3_, NO_2_, O_2_, and CO_2_, has been achieved using hydrogels as gas-sensitive materials. For this application, hydrogels exhibit flexibility and stretchability to ensure the comfort of the users, which realizing real-time and reliable monitoring of the concentration of target gases at room temperature, thereby offering long-term protection for health and safety. In addition, the unique self-healing ability of the hydrogel allows the sensor able to repair itself under mechanical damage during use and extend its applicable life. Thus, hydrogel-based gas sensors have been used in various fields from environmental monitoring, health care, personal safety protection, to smart life, which demonstrate their promising applications. This section reviews the recent research progress of hydrogel-based gas sensors with a brief explanation of their gas-sensitive mechanisms based on existing studies. At the end of this section, the performance parameters of hydrogel-based sensors are compared with those using other materials with NO_2_ and NH_3_ as target gas examples (Table 1) to highlight the advantages and potential of hydrogel as a sensitive material for flexible and wearable gas sensors.

**Table 1:** Sensing performance of hydrogel-based and material based other NO_2_/NH_3_ sensors

Materials	Target	C	Res./Sens	t_res_/t_rec_	LOD	D	Temp	Ref.
S-RGOH	NO_2_ NH_3_	4 ppm 20 ppm	23.5% 7%	12/11 s 16 s	4.1 ppb 1.48 ppm	Bending	RT	[208]
V-RGOH	NO_2_ NH_3_	10 ppm 20 ppm	36.3% 10.1%	284/363 s 149/284 s	0.07 ppm 0.42 ppm	Bending	RT	[209]
SnO_2_/RGOH	NO_2_	5 ppm	32.1%	177/260 s	2.8 ppm	Bending	RT	[210]
PAM/Carr-CaCl_2_	NO_2_	4 ppm	460%	29.8/41.0 s	86 ppt	824% strain	RT	[150]
PAM/CArr-CaCl_2_-Gly	NO_2_	2 ppm	121.1%	79.7/71.3 s	6.8 ppb	1400% strain	RT	[69]
PVA–CNF-Gly	NO_2_	250 ppb	372% ppm^−1^	41/144 s	2.23 ppb	672% strain	RT	[67]
PVA /CA	NH_3_	40 ppm	31.60%	46 s/9 s	140.85 ppb	330.44% strain	RT	[68]
PAM/CArr	NO_2_ NH_3_	500 ppb 40 ppm	78.5 ppm^−1^ 1.3% ppm^−1^	101/46.8 s 225/13 s	1.2 ppb 220 ppb	1200% strain	RT	[126]
PAM/CArr-Eg	NO_2_ NH_3_	5 ppm	8.4 ppm^−1^ 1.1 ppm^−1^	271.9/80.1 s 365/60.1 s	34.9 ppb 91.6 ppb	1325% strain	RT	[70]
PAA/GA	NH_3_	50 ppm	9.2 (Z_a_/Z_g_)	–	120 ppb	Yes	RT	[72]
PGA/GA	NH_3_	50 ppm	8.4(Z_a_/Z_g_)	–	72 ppb	Yes	RT	[71]
CA/PEGDA	NH_3_	20 ppm	6.20(Z_a_/Z_g_)	–	50 ppb	Yes	RT	[76]
MoS_2_	NO_2_	100 ppm	0.35% ppm^−1^	29/350 s	–	No	RT	[251]
SWCNT/PdO/Co_3_O_4_	NO_2_	20 ppm	27.33%	–	1 ppm	Bending	RT	[252]
MWCNT/WO_3_	NO_2_	5 ppm	14%	10/27 min	100 ppt	Bending	RT	[253]
WO_3_ nanoplates	NO_2_	100 ppm	131.75(R_g_/R_a_)	–	5 ppm	No	100 °C	[254]
Au-WO_3_	NO_2_	5 ppm	136(R_g_/R_a_)	4/59 s	< 0.25 ppm	No	100 °C	[255]
rGO/ZnO	NO_2_	50 ppb	12(R_g_/R_a_)	5.1/7.5 min	5 ppb	No	100 °C	[256]
PANI/CeO_2_	NH_3_	50 ppm	262.7%	14/6 min	16 ppb	Bending	RT	[257]
CuO	NH_3_	5 ppm	25%	90/120 s	5 ppm	Bending	RT	[258]
PANI/α‑Fe_2_O_3_	NH_3_	100 ppm	39%	27/46 s	5 ppm	Bending	RT	[259]
Ti_3_C_2_ MXene	NH_3_	500 ppm	6.13%	45/94 s	10 ppm	No	RT	[260]
Ti_3_C_2_T_x_ MXene	NH_3_	100 ppm	0.8%	–	100 ppb	No	RT	[44]

C: Concentration; Res./Sens.: response or sensitivity; t_res_/t_rec_: response time/recovery time; D: deformability; Temp.: operating temperature; RT: room-temperature; Z_a_: the impedance modulus in the air; Z_g_: the impedance modulus in the target gas; R_a_: sensor resistance in air; R_g_: sensor resistance in target gas

### Gas-Sensing Mechanism

Compared with traditional semiconductor- and electrochemical-based gas sensors, conductive hydrogel-based gas sensors are still in its infancy, and their mechanisms are still unclear. Nonetheless, various studies are trying to unveil the mechanism behind hydrogel gas sensing, which have made a significant contribution to the clarification of their sensing mechanisms.

**Fig. 6:**
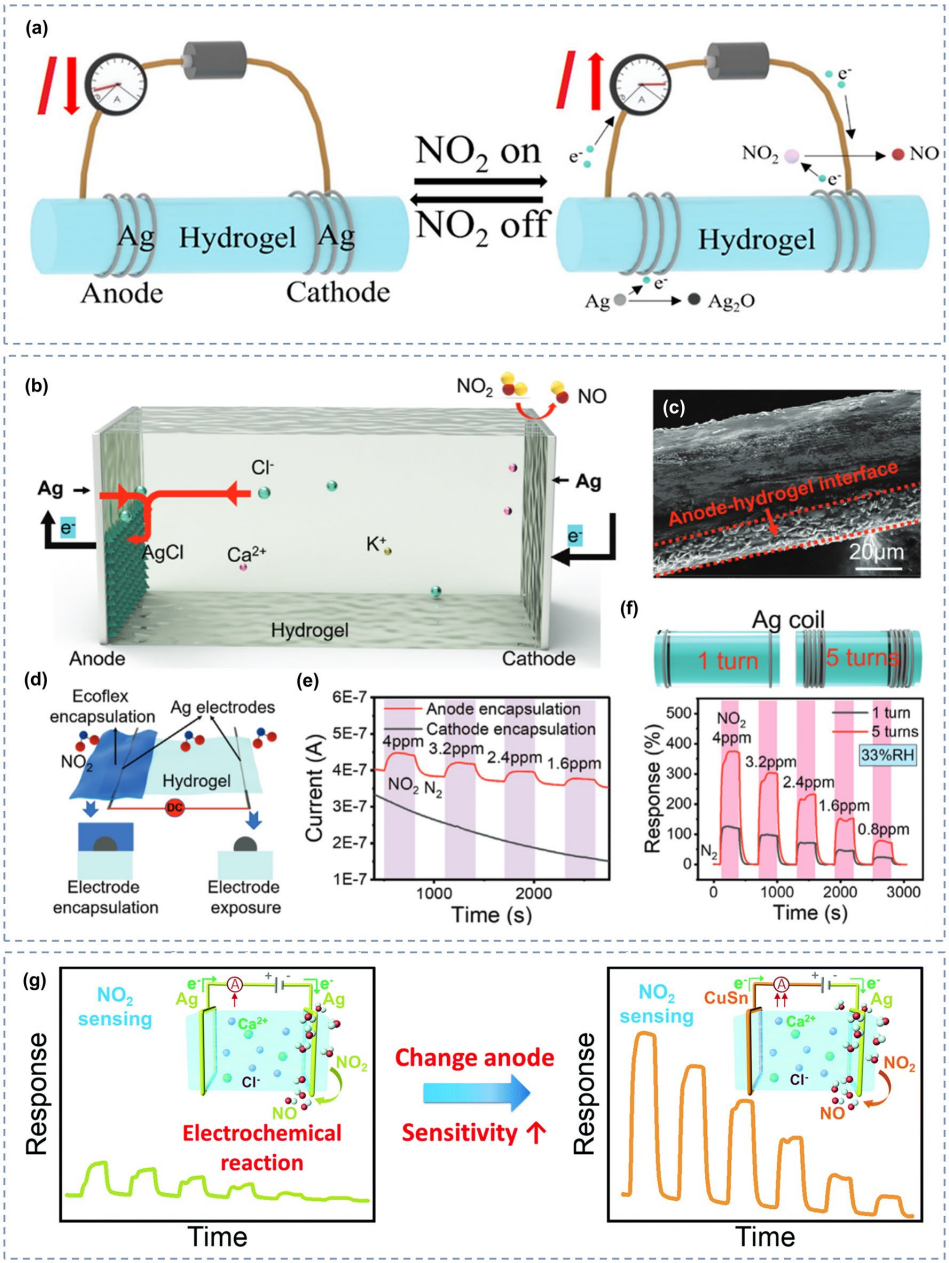
**a** Schematic of a simple hydrogel-based gas sensor and its sensing process. Reproduced with permission [67]. Copyright 2023, Wiley‐VCH. **b** Schematic of the sensing mechanism of hydrogel for NO_2_ sensing. **c** SEM image of the Ag anode after prolonged NO_2_ sensing, indicating the generation of an AgCl layer. **d** Schematic of the electrode-masking experimental setup. **e** Real-time current curves of the hydrogel sensor for different NO_2_ concentrations when the anode or cathode is encapsulated. **f** Dynamic response curves of the hydrogel sensors with different numbers of Ag electrode turns to different NO_2_ concentrations. Reproduced with permission [150]. Copyright 2021, Wiley‐VCH. **g** Effect of anode material change on the sensitivity of hydrogel-based NO_2_ sensors. Reproduced with permission [69]. Copyright 2022, Royal Society of Chemistry

Hydrogel-based gas sensors can function as essential electrochemical sensors. As shown in the Fig. 6a, a simple gas sensor is formed by adding one electrode at the ends of a hydrogel. In this case, the hydrogel serves as an electrolyte that carry different conductive particles, whereas the electrodes at each end are the sensitive (working electrode) and counter electrodes, respectively. A constant DC voltage is applied at the two electrodes, and the current and other electrical parameters between them are monitored in real time. As the sensor is exposed to a certain concentration of the target gas, a redox reaction occurs between the gas molecules and electrode at the interface of the sensitive electrode and hydrogel. Depending on the target gas, electrons are released to or captured by the electrode, resulting in a change in the current or other electrical parameters between the electrodes, thereby generating a response signal. The amplitude of the response signal further changes with the concentration of the target gas.

Recent studies have validated the sensing process of hydrogel gas sensors [67, 69, 150, 206]. Take hydrogel-based NO_2_ gas sensor as an example, Wu et al. [150] elaborated its sensing mechanism and further verified it through experiments. As shown in Fig. 6b, the response reaction of NO_2_ occurs at the cathode-hydrogel junction, where NO_2_ is reduced and OH^−^ ions are consequently produced in the hydrogel with the same number of consumed electrons, thereby increasing the current. In addition, the Ag electrode is oxidized (anode) to produce AgCl, which allow more electrons and ions to participate in the conduction and increase in current. To verify this conjecture, they designed an electrode-masking experiment, as shown in Fig. 6d. The cathode or anode electrodes were separately covered, and their respective response change curves were collected (Fig. 6e). If the anode is covered, the sensor continuously responds, whereas if the cathode is covered, the sensor does not produce sensing signals for NO_2_. Further, after the response reaction, the Ag electrode was observed under a scanning electron microscope (SEM). Figure 6c shows an additional layer of AgCl added to the anode electrode, which reflects the reliability of the previous deduction from the side. In addition, they investigated the effect of the number of Ag electrode turns on the NO_2_ response. Under the same temperature and humidity, the NO_2_ response of the sensor with five turns of the Ag electrode was significantly greater than that with one turn, as depicted in Fig. 6f. This result confirms the significant influence of the electrodes on the sensing reaction of hydrogel-based gas sensors, as an electrochemical reaction.

To further investigate the effect of the electrodes on the sensing performance of hydrogel-based gas sensors, Wu et al. [69] tested the NO_2_ response of different electrode materials on the same hydrogel substrate in another study. With Ag as the cathode material and Ag or CuSn as the anode material, they tested the change of the sensor response to NO_2_ under different anodes, respectively. Figure 6g illustrates the increased sensitivity from 31.18 to 60.02% ppm^−1^ when anodic Ag was replaced with CuSn alloy owing to the stronger oxidation tendency of Cu and Sn; thus, the electrode material has a strong influence on the gas-sensing performance. In addition to the increased sensitivity, the CuSn anode displays better corrosion resistance than that with the Ag anode, leading to a prolonged lifespan.

**Fig. 7:**
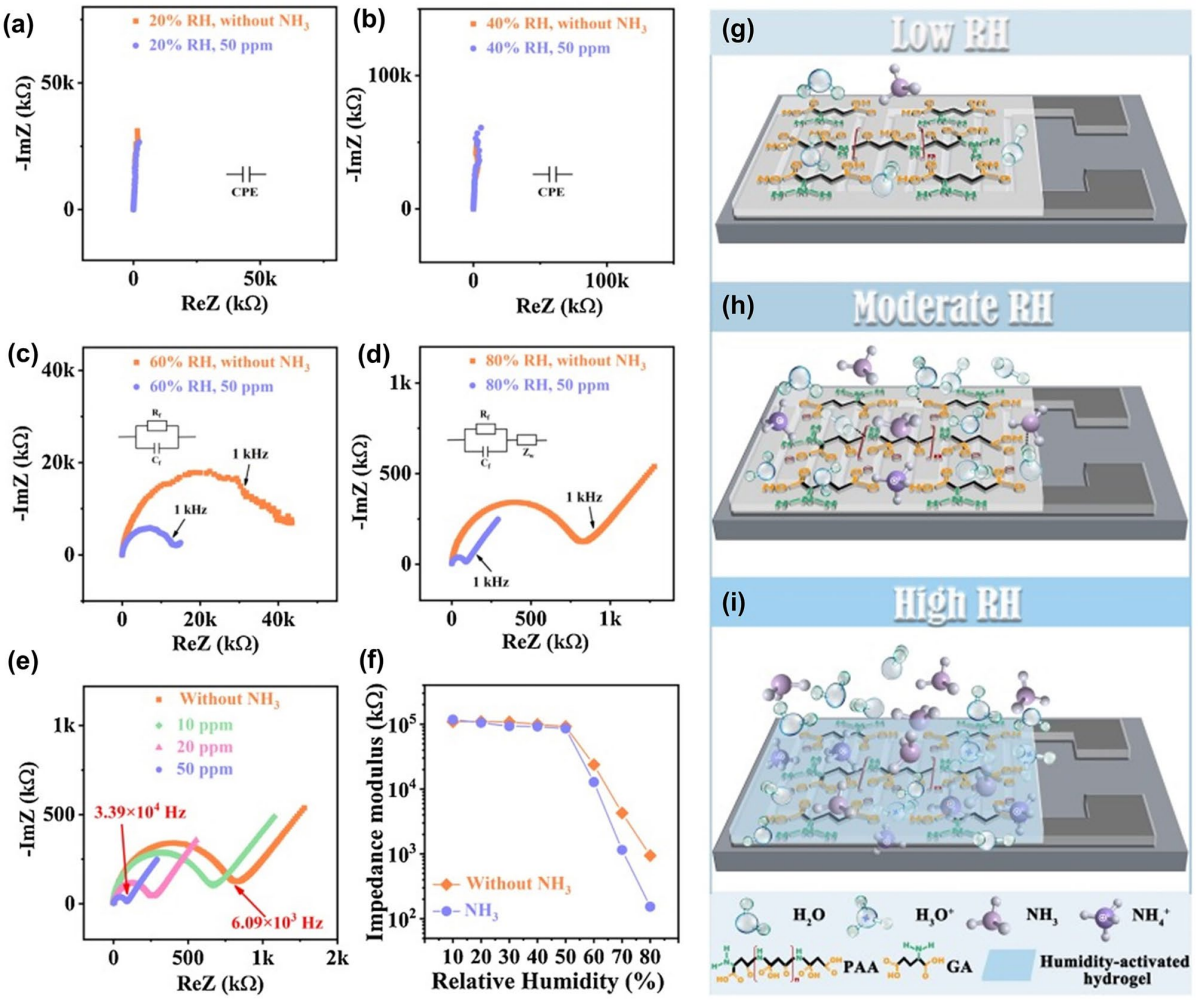
**a-d** Complex impedance plots of the PAA/GA sensor at different humidity values with or without 50 ppm NH_3_. **e** Complex impedance plot of the PAA/GA sensor with different NH_3_ concentrations at 80% RH. **f** Impedance modulus of the PAA/GA sensors with and without 50 ppm NH_3_ at different humidity levels. NH_3_ sensing mechanism of PAA/GA: **g** at low RH values, **h** at moderate RH values, and **i** at high RH values. Reproduced with permission [72]. Copyright 2021, Elsevier

Ambient humidity has a great impact on the response characteristics of hydrogel-based gas sensors. In low-humidity environments, the conductive channels in the hydrogel are not connected, and the sensing loop is equivalent to that of a broken circuit, which prevents further response reaction with the target gas. As the ambient humidity is gradually increased, the conductive pathway in the hydrogel is connected, and the target gas can further react with the hydrogel and electrode. Zhang et al. [72] explored the role of RH in the sensing properties of hydrogel-based gas sensors. Taking NH_3_ as an example, they used poly-_L_-aspartic acid (PAA)/_L_-glutamic acid (GA) composite hydrogels as the research object. The hydrogels were placed in environments with different humidity and NH_3_ concentrations, and their complex impedance maps were tested from 20 Hz to 20 MHz at 1 V. Their corresponding equivalent circuits were provided. At low-humidity values (10–50% RH; Fig. 7g), no proton-conducting channels were noted in the hydrogel-sensitive film, and its impedance did not change significantly even with the introduction of NH_3_ (Fig. 7a–b). At moderate humidity values (Fig. 7h), hydrophilic groups, such as carboxyl, amino, and amide groups, absorb water molecules to form a hydrogen bonding network and proton conduction pathways. The introduced NH_3_ can combine with water molecules through hydrogen bonding, which are adsorbed on the sensitive membrane, thereby affecting the impedance of the device (Fig. 7c). At high-humidity values (Fig. 7i), the hydrophilic hydrogel-sensitive membrane adsorbs more water molecules and is activated. The water molecules are self-ionized into H^+^ and OH^−^, with some of the H^+^ ions combining with other water molecules to form H_3_O^+^ under the grotto mechanism (Eq. 1). In addition, the sensitive membrane exhibits ionic conductivity. When NH_3_ is introduced, the hydrogel-sensitive membrane takes up a large amount of NH_3_ by acid–base adsorption. On the one hand, H_2_O reacts with the dissolved NH_3_ to form NH_4_^+^ under a proton transfer reaction (Eq. 2). Furthermore, the carboxyl group in GA reacts with NH_3_ to form carboxylate and ionizes into NH_4_^+^ and RCOO^−^ in the liquid phase (Eq. 3). Under the combined effect of multiple components, the resistance of the hydrogel-sensitive membrane decreases, and its conductivity increases, producing a strong response signal (Fig. 7d). Therefore, humidity is a key factor for the NH_3_ adsorption and response of hydrogels. In particular, as the humidity increases, the NH_3_ adsorption, and production of NH_4_^+^ and OH^−^ ions increases, thereby achieving more pronounced changes in the conductance of the hydrogel.

1 $$ H_{2} O(ads) + H_{2} O(ads) \rightleftharpoons H_{3} O^{ + } + OH^{ - } $$

2 $$ NH_{3} (ads) + H_{2} O(ads) \rightleftharpoons NH_{4}^{ + } + OH^{ - } $$

3 $$ RCOOH + NH_{3} (ads) \to NH_{4}^{ + } + RCOO^{ - } $$

The research on the response mechanism of hydrogel-based gas sensors is still in its initial stage. Thus, the effects of the electrode materials, properties of the hydrogel, and external environment on the gas response of the hydrogel need to be further investigated by designing reliable experiments. We are anticipating the increased study on this aspect with more researchers joining the exploration and investigation of the hydrogel gas-sensing mechanisms in the future.

### NO_2_ Gas Sensors

**Fig. 8:**
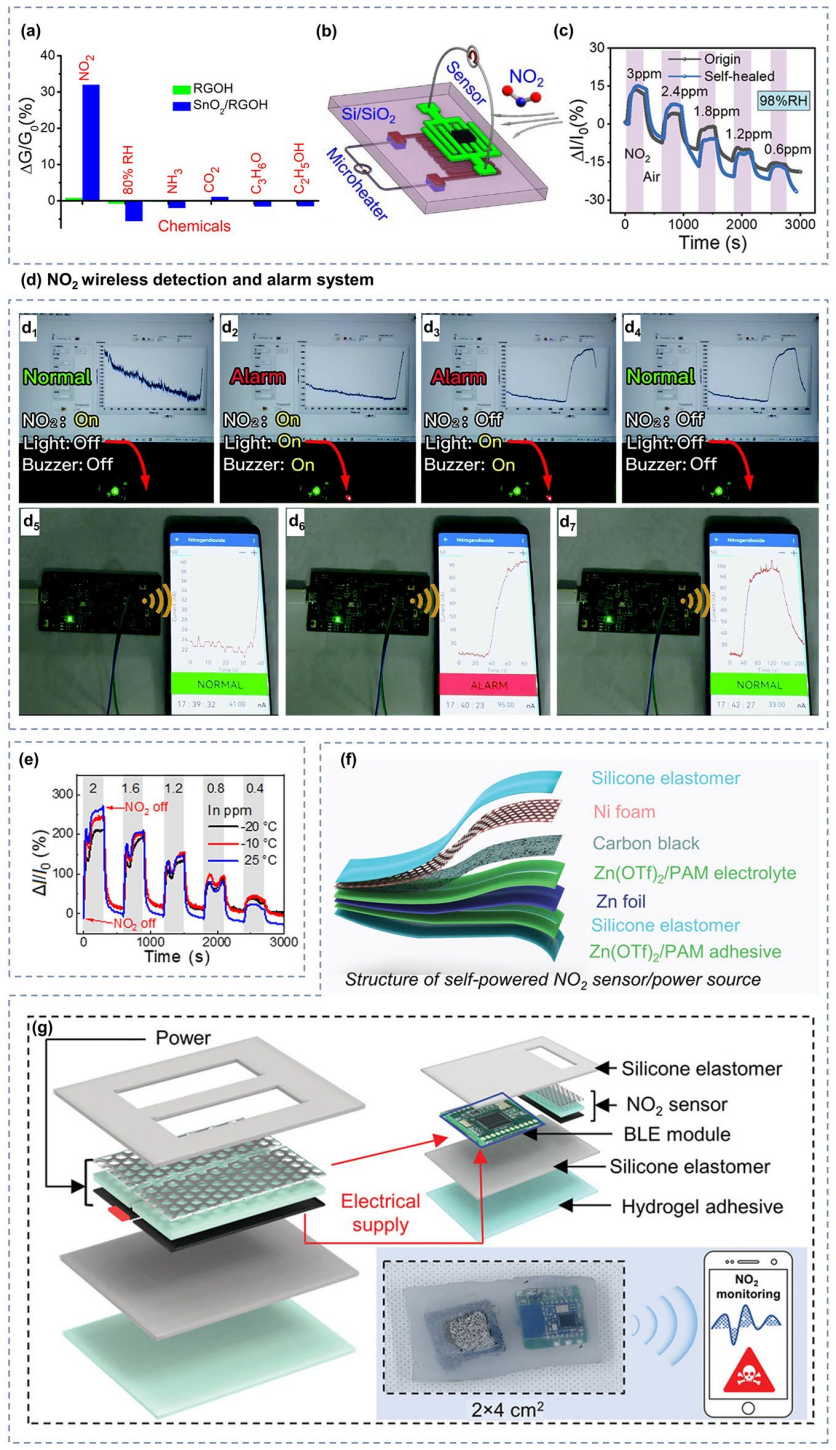
**a** Schematic diagram of the selectivity of SnO_2_/RGOH for NO_2_. **b** Diagram of microheater. Reproduced with permission [210]. Copyright 2020, American Chemical Society. **c** Comparison of the response ability of the original hydrogel and the self-healed hydrogel to NO_2_. Reproduced with permission [150]. Copyright 2021, Wiley‐VCH. **d** Schematic of wireless sensing and alarm for NO_2_. **d**_**1**_**-d**_**4**_ Sensor alarm process for 2 ppm NO_2_: when the current exceeds the threshold, the red light comes on and the buzzer sounds. **d**_**5**_**-d**_**7**_ Wireless reception of NO_2_ sensing signals on a smartphone. Reproduced with permission [69]. Copyright 2022, Royal Society of Chemistry. **e** Real‐time responses of the PVA–CNF DN organohydrogel towards 0.4–2 ppm NO_2_ at 25, − 10 and − 20 °C. Reproduced with permission [67]. Copyright 2022, Wiley‐VCH. **f** Structure of self-powered NO_2_ sensor/power source. **g** Schematic diagram of self-powered NO_2_ sensing system. Reproduced with permission [214]. Copyright 2023, Wiley‐VCH

NO_2_ is one of the most studied gases in current hydrogel-based gas sensors. It is a reddish-brown gas with an irritating odor and is often produced by burning fossil fuels. It is extremely toxic, as little as 1 ppm is sufficient to cause headaches, breathing problems or to cause eye, nose or throat infections. When the concentration of NO_2_ exceeds 20 ppm, it can even be directly harmful to human health [207]. Therefore, it is significant to develop high-performance and low-cost wearable NO_2_ sensors to protect people’s daily health and safety. RGOH is an electronically conductive hydrogel material with a three-dimensional porous structure, and its large specific surface area enables it to adsorb more gases, which is very favorable for gas sensing. With the aim of real time monitoring of NO_2_ and NH_3_, Wu et al. [208] developed a hydrogel-based gas sensor with detection limit as low as 4.1 ppb NO_2_ and 1.48 ppm NH_3_, respectively. They use reduced graphene oxide hydrogels modified with NaHSO_3_ to form a sulfide one (S-RGOH). Compared with the unmodified device, the response of S-RGOH to NO_2_ and NH_3_ was improved by 118.6 and 58.9 times, respectively. Nevertheless, considering the applications of portable and wearable gas sensors, the material composition of the sensors should be green, non-toxic and biocompatible. In order to guarantee that the sensor itself should not cause harm to the human body during usage, green and non-polluting substances that are harmless to human beings could be chosen to modify the hydrogel, so that the sensing performance can be improved while protecting human health and safety. Wu and his team [209] improved their previous study by choosing to use green, non-polluting vitamin C (VC) as a modifier for the reduced graphene oxide hydrogel (V-RGOH). The sensor maintains good performance while being green and harmless. The response of V-RGOH devices was 52.5 and 36.3% for 100 ppm NH_3_ and 10 ppm NO_2_ with detection limits as low as 0.42 and 0.07 ppm, respectively. The strong interaction between VC and the measured gas, and the combination of multiple active sites due to the three-dimensional porous structure, improve the sensing performance of RGOH for NH_3_ and NO_2_. Whereas the multiple sensitivities to NO_2_ and NH_3_ still affect the selectivity of the hydrogel for each of the two gases. Further enhancements are needed to improve the selectivity of hydrogel-based gas sensors for NO_2_ and NH_3_, respectively. The potential of metal oxide semiconductor (MOS) materials for gas sensing is well documented, and although most MOS-based sensors need to operate at relatively high temperatures, the excellent sensitivity and selectivity they exhibit for a wide range of gases is very promising. Therefore, combining metal oxides with RGOH solves the problem of high-temperature operation while improving the overall response and selectivity of the sensor. To further increase the selectivity of hydrogel-based gas sensors for NO_2_, Wu et al. [210] prepared a three-dimensional graphene hydrogel modified with SnO_2_ (SnO_2_/RGOH). The SnO_2_/RGOH NO_2_ sensor has a very low theoretical detection limit of 2.8 ppm and a high sensitivity of 4.3 ppm^−1^ at room temperature. Moreover, as illustrated in Fig. 8a, the selectivity of the sensor for NO_2_ is greatly enhanced by the increased sensitivity of the device to NO_2_ and the improved conductivity of the material due to the presence of the p–n junction between RGOH and SnO_2_.

However, in order to keep these sensors undisturbed by humidity and temperature changes at room temperature, the authors in all three studies mentioned above integrated a micro-heating device outside the hydrogel, as depicted in Fig. 8b. Although this device can improve the stability of the hydrogel-based gas sensor to some extent, it brings higher preparation cost and difficulty, as well as increases the power consumption and safety risk during practical use. A more convenient and safe approach is to introduce hygroscopic salts into the hydrogel to enhance its gas-sensitive response properties while improving its water-retaining ability and stability. Wu et al. [150] developed a salt infiltrated hydrogel that can realize real-time monitoring of NO_2_ at room temperature without any other treatments. They used a simple solution infiltration method to incorporate CaCl_2_ into the PAM/Carr DN hydrogel. In the presence of CaCl_2_, this hydrogel NO_2_ sensor exhibited a high sensitivity of 119.9%, short response and recovery time (29.8 and 41.0 s, respectively), good linearity, low theoretical limit of detection (LOD) of 86 ppt, high selectivity, stability, and ionic conductivity. The sensor is self-healing as it recovers after being truncated and does not affect the conductive and sensitive properties, which means it is more tolerant, as shown in Fig. 8c. Moreover, the sensitive characteristics of the sensor are not affected by tensile deformation, and it can maintain the sensing of NO_2_ even at 100% strain with shortened response and recovery time. Wei et al. [69] also prepared a NO_2_ gas sensor operating at room temperature using PAM/CArr DN hydrogel with active metal electrodes. CaCl_2_ and Gly were introduced to improve its conductivity and mechanical deformability as well. The sensor can not only withstand 1,400% tensile strain due to its unique DN structure, but also exhibits a high sensitivity of 60.02% ppm^−1^ and the ultralow LOD of 6.8 ppb NO_2_. The sensor also features excellent selectivity for NO_2_ and still works well at 50% tensile strain. Even further, the authors implemented wireless sensing of NO_2_ by collecting and processing data from real-time monitoring. When the concentration of NO_2_ exceeds the threshold, both the smartphone and the alarm system will give an early warning, as shown in Fig. 8d.

Nonetheless, the operation of hydrogel gas sensors in extreme environments still presents challenges. For example, in environments where the temperature is too high or too low, there is a risk of evaporation or freezing of the water in the hydrogel that prevents it from working properly for detection. The introduction of organic polyol into hydrogel can solve the problem to some extent. For example, Gly has a lower freezing point than water, and the hydroxyl groups in it combine with surrounding free water molecules through hydrogen bonding, which effectively inhibits the formation of hydrogen bonds between water molecules at sub-zero temperatures, and directly preventing ice crystal formation and lowering the freezing point [211–213]. Ding et al. [67] developed a NO_2_ hydrogel sensor capable of operating at room temperature or even at − 20 °C, as illustrated in Fig. 8e. They used a PVA–CNF DN organohydrogel with a water/Gly binary solvent to realize high-efficiency sensing of NO_2_. The sensor has ultrahigh sensitivity (372% ppm^−1^), low LOD (2.23 ppb), fast response and recovery time (41/144 s for 250 ppb NO_2_), and high selectivity to NO_2_. After being cut or remolded, it can still heal, recover and retain its original conductive and sensitive properties perfectly, which implies better endurance and more application possibilities, demonstrating the potential of the sensor for actual use.

Nevertheless, all of the above NO_2_ sensors require additional power supply devices to provide energy for their operation, and the rigidity and bulkiness of traditional external power supplies limit the overall nature of the flexible vapor sensor, sacrificing advantages such as convenience and comfort. Thus, the development of self-powered vapor sensor is of great practical importance. Since the hydrogel-based gas sensor is based on electrochemical sensing, an electrochemical cell can be coupled to the hydrogel-based gas sensor to achieve self-powered sensing. Wu et al. [214] constructed a novel self-powered flexible NO_2_ sensor using the structure of Zn-zinc trifluoromethanesulfonate (Zn(OTf)_2_)/PAM hydrogel-Carbon, as demonstrated in Fig. 8f. The sensor has ultra-high sensitivity (1.92% ppb^−1^), very low theoretical LOD (0.1 ppb) and excellent NO_2_ selectivity. It works even in complex environments such as stretching, bending, sub-zero, and high humidity environment. The authors also assembled it with miniaturized circuit modules, as shown in the Fig. 8g, and successfully built a self-powered integrated wireless NO_2_ monitoring system for real-time and remote gas detection.

### NH_3_ Gas Sensors

Ammonia is another toxic and harmful gas from human excretion and industrial production. Although it has a pungent odor, it is easily detectable only when its concentration is higher than 50 ppm [215]. It can scorch the skin, eyes, and mucous membranes of respiratory organs, and if people inhale too much, it can cause lung swelling and even death. Only more than 35 ppm of NH_3_ is sufficient to potentially destroy the human sensory and respiratory systems [216, 217]. Ammonia is widely used in the electronics industry, food processing, chemical and scientific research fields. Thus, real-time monitoring of the presence and concentration of NH_3_ is necessary to ensure human safety. In addition, ammonia is one of the gases in the body’s exhaled breath that can be used to mark the presence of a certain disease [218]. Generally, the concentration of NH_3_ in human exhalation is roughly between 0.5 to 2 ppm. When the concentration increases, it indicates possible kidney failure, liver dysfunction, peptic ulcer and other diseases. While when the concentration decreases, it indicates possible asthma [218–220]. In general, whether in protecting the human body or health monitoring, we require NH_3_ sensors with excellent performance to protect our wholesome life.

**Fig. 9:**
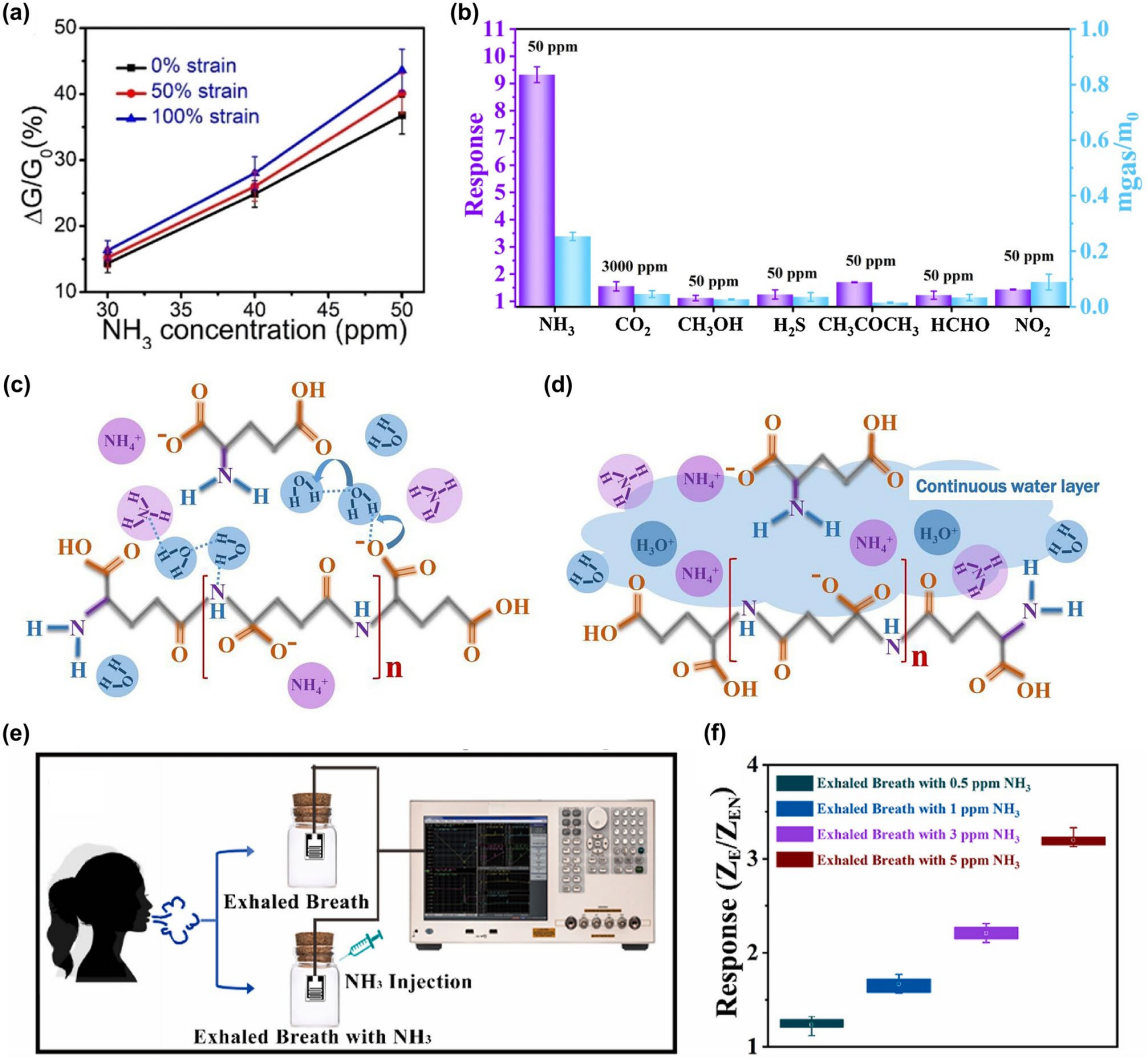
**a** Response of PAM/CArr DN hydrogel to NH_3_ in different tensile strains. Reproduced with permission [70]. Copyright 2020, American Chemical Society. **b** Response (purple column) of PAA/GA sensor to NH_3_ and other interfering gases. Reproduced with permission [72]. Copyright 2021, Elsevier. **c-d** NH_3_ sensing mechanism of PGA/GA sensors: **c** at low RH values and **b** at high RH values. Reproduced with permission [71]. Copyright 2020, Elsevierc.** e** Diagram of the breath testing process for NH_3_. **f** Response of hydrogels to exhalation with different NH_3_ contents. Reproduced with permission [76]. Copyright 2021, Elsevier

Recently, it has been shown that oxygen-containing functional groups such as − OH, SO_3_^−^and − NH_2_ play a non-negligible role in promoting the adsorption of NO_2_ and NH_3_ molecules, so functionalized hydrogels such as PVA and PAM rich in oxygen-containing groups are also commonly used in the preparation of gas sensors [221, 222]. Zhi et al. [68] prepared NH_3_ and NO_2_ hydrogel gas sensors based on PVA and CA double networks with the response of 31.60%, response/recovery times of 46/9 s for 40 ppm NH_3_ and low LODs of 140.85 ppb while being stretchable. Further, in order to make the hydrogel have stronger mechanical deformability as well as better water storage capacity for complex practical application environments, Wu and his team [126] introduced the Gly into chemically derived ion-conductive PAM/CArr DN hydrogels to prepare a high-performance stretchable room-temperature NH_3_ and NO_2_ sensor. It can not only withstand various stringent mechanical deformations, including up to 1.200% strain, wide ranges of bending and torsion, but also has high sensitivity (78.5 ppm^−1^ to NO_2_ and 1.3 ppm^−1^ to NH_3_) and low LOD (1.2 ppb for NO_2_ and 220 ppb for NH_3_). And the gas-sensitive performance of the sensor will not be impaired by the strong mechanical deformation, which means that the sensor will not fail to work properly in practical wearable applications due to human movement or other possible stretching scenarios. Afterwards, they introduced ethylene glycol (Eg) modification on the basis of PAM/CArr DN hydrogel to prepare NH_3_ and NO_2_ hydrogel gas sensors that can operate at room temperature [70]. The sensor has high sensitivity to NH_3_ (1.4 ppm^−1^), low LOD (92 ppb), and retains sensitive characteristics under various mechanical deformations (Fig. 9a). In addition, it has extremely high resistance to drying and freezing (avoid drying within a year and its freezing point is below − 130 °C), high transparency and self-healing ability, enabling adaptation to a wider range of practical environments and application situations.

However, the above gas sensors are both NH_3_ and NO_2_ sensitive, it is difficult to exclude the interference of NO_2_ gas on the response signal in practical applications, and the research and development of gas sensors with high NH_3_ selectivity are warranted. NH_3_ has a relatively high solubility in water, so increasing the ambient humidity and consequently the content of water molecules in the hydrogel can effectively improve the adsorption of NH_3_ molecules and enhance the response of the sensor to NH_3_. Liu et al. [72] developed an environmentally friendly and non-toxic biomass hydrogel-based NH_3_ sensor employing PAA and GA as sensing materials based on a humidity-activated mechanism at room temperature. The sensor has a response of 9.2 to 50 ppm NH_3_ at room temperature with 80% RH. The PAA/GA hydrogel has better sensing characteristics for NH_3_ in high humidity environments, especially its selectivity (Fig. 9b). The detailed mechanism has been mentioned above. In addition, they made a test on human exhalation, the sensor was able to accurately identify the presence of 5 ppm NH_3_ in the exhaled gas, which means that the sensor has the potential to be used in healthcare applications.

Similarly, Liu et al. [71] prepared a highly sensitive, non-toxic, and environmentally friendly electrical NH_3_ hydrogel sensor under humid environment at 25 °C using biomass hydrogel GA and poly-_L_-glutamate (PGA) as sensitive composite materials. The sensor showed a response of up to 8.4 to 50 ppm NH_3_ at 80% RH with a LOD as low as 0.5 ppm, and good NH_3_ selectivity. And the high response performance of PGA/GA mainly comes from the synergistic effect unique to PGA and GA. As shown in Fig. 9c-d, firstly, PGA and GA have good chemical compatibility because of their similar chemical structures. Secondly, the appropriate amount of PGA in PGA/GA enables the hydrogel to maintain a certain adsorption capacity for water and NH_3_. As mentioned above, at low humidity, the adsorbed water will form a hydrogen bonding network with some groups available for proton leap (grotto mechanism), when the device is in weak proton conduction. At high humidity, the sensitive material adsorbs more water to form a condensed water layer, and the proton conductivity makes H_2_O and NH_3_ start to move. The adsorbed NH_3_ easily forms ammonium salts with some groups, and the presence of NH_4_^+^ and H_3_O^+^ ions on the surface of the material and the water layer enhances the mobility of these ions in water, which greatly increases the conductivity of the sensor. In addition, during sensing, NH_3_ is physically adsorbed and dissolved in the ligand water, generating NH_4_^+^ ions. And carboxylic acid groups are rich in GA and PGA, which can also react with NH_3_ to generate NH_4_^+^. Therefore, the PGA/GA hydrogel NH_3_ sensor has better response characteristics in high humidity environments.

Zhang and her team [76] used thiol-ene photochemistry to encapsulate citric acid (CA) into crosslinked polyethylene glycol diacrylate (PEGDA) hydrogels to prepare a highly sensitive and selective CA/PEGDA hydrogel-based NH_3_ gas sensor working at high humidity (80% RH). The sensor exhibited a high response (6.20) to 20 ppm NH_3_ at room temperature, as well as a low LOD (50 ppb). This is due to the equilibrium water content of the hydrogel composite and the moderate acid dissociation constants of the acid groups. This sensor exhibits good chemical stability, while the stable hydrogel structure facilitates the detection of NH_3_ in humid environments. Specifically, CA has an extremely strong hydrophilicity and NH_3_ adsorption ability. At 80% RH, the large amount of adsorbed water enhances the activity of the carboxyl group, which causes the carboxyl group to react with a large amount of adsorbed NH_3_ to form ammonium salts. The ammonium salt has stronger hydrophilicity than the carboxyl group, and the adsorption of water increases, leading to CA membrane deliquescence. Unlike CA, PEGDA exhibits relatively poor hydrophilicity, and the amount of NH_3_ adsorbed is relatively small even at higher moderate environments, blocking the formation of carboxylates. Compounding CA with PEGDA allows the hydrogel to maintain a good NH_3_ adsorption capacity while still possessing an appropriate hydrophilicity. As a result, CA/PEGDA has both high NH_3_ response and good reproducibility. In addition, the carboxyl group in the CA structure can provide more adsorption sites for NH_3_, thus, the CA/PEGDA composite has good response properties to NH_3_ at 80% RH. They also tested for the presence of NH_3_ in human exhalation, the sensor can accurately identify different concentrations of NH_3_ in the exhaled breath, as shown in Fig. 9e-f, demonstrating the potential for clinical application of the sensor.

However, the hydrogel NH_3_ sensors described above, especially the last three examples, typically have a higher response in high humidity environments. Although this feature is more friendly for breath detection applications, there is a problem of low response for NH_3_ monitoring in other situations. Subsequent development of hydrogel-based gas sensors with high sensitivity and selectivity for NH_3_ at room temperature and normal RH or even low RH should be continued to adapt to more complex application scenarios.

### O_2_ Gas Sensors

**Fig. 10:**
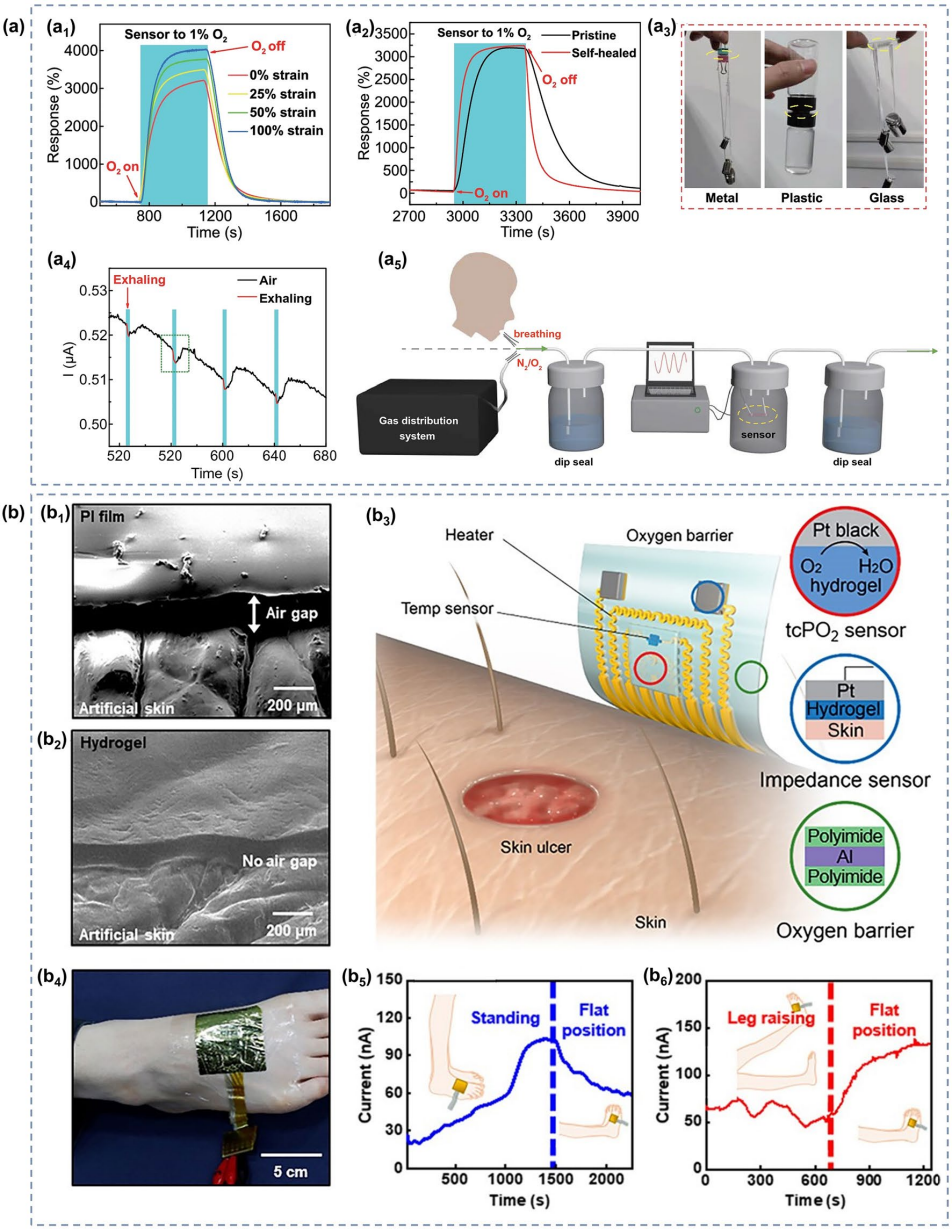
**a**_**1**_ Response to 1% O_2_ under different tensile strains. **a**_**2**_ Response of original and self-healed organohydrogels to 1% O_2_. **a**_**3**_ Photographs showing the organohydrogel (marked by the yellow circle) adhered firmly to various materials. **a**_**4**_ Real-time response curve of the sensor to human exhalation in the air. **a**_**5**_ Diagram of the breath testing process for O_2_. Reproduced with permission [77]. Copyright 2022, Springer Nature. SEM of **b**_**1**_ PI film and **b**_**2**_ hydrogel on artificial skin. **b**_**3**_ Schematic diagram of the tcPO_2_ sensing device and sensing process. **b**_**4**_ Illustration of placing the sensor on the foot to measure tcPO_2_. **b**_**5**_**-b**_**6**_ Test results of tcPO_2_ sensing when the foot is in different states. Reproduced with permission [230]. Copyright 2021, American Association for the Advancement of Science

Oxygen is an essential gas that is indispensable for the survival of living things. It is involved in the aerobic decomposition of carbohydrates that provide energy for life activities [223]. When the concentration of oxygen is below 17%, it causes adverse physiological reactions such as difficulty in breathing and weakness. When the oxygen concentration is below 12%, there is even a risk of death by asphyxiation [224, 225]. Nevertheless, too high oxygen concentration is not a good thing. When people are exposed to high partial pressures of oxygen (PO_2_), the central nervous system and the pulmonary system will suffer damage, some of which is even irreversible [226–228]. However, sometimes a high oxygen concentration environment is still desirable, such as anesthesia machines and hyperbaric chambers in hospitals [224, 229]. In order to avoid the danger of oxygen poisoning or hypoxia, a monitoring of the oxygen concentration is required, and it is necessary to develop sensor devices that can monitor oxygen concentration over a wide range. Liang et al. [77] prepared a room-temperature O_2_ sensor (in an oxygen-free environment) using a PAM-CS DN structured organohydrogel. It is capable of monitoring the full O_2_ concentration range with a theoretical minimum detection limit as low as 5.7 ppm, sensitivity as high as 0.2% ppm^−1^, and good resistance to temperature and humidity. In addition, the PAM-CS organohydrogel sensor has stretchability, which allows sensing even under 100% stretching without the loss of sensing performance (Fig. 10a_1_); self-healing capability, which can self-heal and compound even after being cut and restore the original response characteristics (Fig. 10a_2_); and self-adhesive capability, which allows the adhesion on the skin surface without the use of additional binders (Fig. 10a_3_). Since the oxygen concentration in exhaled breath is lower than that in air, human respiration can be identified by changes in oxygen concentration. As shown in Fig. 10a_4_–a_5_. The sensor is sensitive to human exhalation, indicating the possibility of its application for respiratory monitoring.

In parallel to respiratory monitoring, oxygen sensors could also be used in other medical applications such as cell culture experiments or monitoring of transcutaneous oxygen pressure (tcPO_2_). When the temperature of the skin surface rises, oxygen diffuses out from the capillaries into subcutaneous tissues and the skin surface. At this time, monitoring the oxygen partial pressure of the skin can reflect the actual oxygen supply to the tissue cells, thus directly reflecting the microcirculation of the skin and indirectly reflecting the macrovascular condition [231, 232]. Liebisch et al. [233] prepared a Clark-type oxygen microsensors that can operate in an aqueous environment. They used a PHEMA hydrogel layer containing a buffer solution as the electrolyte, platinum as the electrode material for the sensor, and polydimethylsiloxane (PDMS) as the gas permeable membrane. They also monitored oxygen in monolayer cultures with T-47D breast cancer cells, which further demonstrates the applicability of the sensor in cell culture oxygen concentration detection. In terms of oxygen sensors for tcPO_2_ monitoring, it is also possible to monitor physiological changes in the human body, such as blood flow rate and the amount of local oxygen supplied to the tissues. Kim and his team [230] propose a skin device interface with tissue-like characteristics that is formed by using an ultrathin type of functionalized hydrogel (PAM). Its softness and mechanical deformability allow it to adhere well to human skin as shown in Fig. 10a-b, while the combination of a highly porous three-dimensional (3D) network and the super-low thickness of the interfacial hydrogel allows for rapid diffusion and transport of the target bioanalyte. Then the authors fabricated an electrochemical device with three electrodes (Pt as working and counter electrode and Ag/AgCl as reference electrode) for the detection of tcPO_2_. To prevent the oxygen from circulating inside and outside the device, they further encapsulated the sensor with a multilayer oxygen barrier film made of PI/Al/PI (polyimide). As depicted in Fig. 10c, measurements are performed by first applying gentle heating to the skin across a hydrogel interface through which oxygen molecules are extracted from the blood vessels under the skin to reach the device. The diffused oxygen is then electrochemically reduced at the working electrode, and the change in reduction current is measured to obtain information about tcPO_2_. By connecting this sensor to the human foot (Fig. 10d), it is capable of detecting the reduction current and tcPO_2_ at different foot positions, with a trend between them roughly as illustrated in Fig. 10e-f. However, in order to obtain more accurate data, more detailed research is needed in the future, such as improving electrode fabrication and measurement protocols.

### CO_2_ Gas Sensors

CO_2_ is ubiquitous and can be produced whenever carbonaceous materials are burned, and as a by-product of respiration, CO_2_ will inevitably be produced as long as organisms are still breathing. The concentration of CO_2_ should not be higher than 1,000 ppm in human working and living environments [234]. Exceeding CO_2_ levels may not only have adverse effects on human health, such as headaches, fatigue, eye diseases, nasal, or respiratory diseases, but also bring about the greenhouse effect and global warming [235, 236]. However, CO_2_ has neither color nor odor, so it is difficult for human-being to detect its presence through the human sensory system, let alone know its concentration. Hence, portable and wearable gas sensors are needed to help people detect the presence and concentration of CO_2_ in their lives and work, and prevent unknown risks.

One way to measure CO_2_ is to first inject a gas containing CO_2_ into an aqueous solution and reflect the amount of CO_2_ by measuring the concentration of dissolved CO_2_. Wang et al. [73] prepared hydrogel membranes from branched polyethyleneimine (BPEI) and partially oxidized dextran (PO-DEX) by in situ Schiff base reaction to achieve rapid, real-time, and continuous monitoring of dissolved CO_2_. The BPEI/PO-DEX films swell in water, and the swelling will be greater when CO_2_ is introduced to react with the amino group in BPEI. The movement of the Fabry–Perot stripe on the reflectance spectrum of the BPEI/PO-DEX film is used as a sensing signal to allow sensitive detection of dissolved CO_2_. In the similar vein, Li et al. [237] developed a gas sensor for detecting dissolved CO_2_ concentration. They prepared a hydrogel sensing grating by replicating a photoluminescent surface grating on a thin film of azo molecular glass on the surface of a hydrogel using soft lithography to achieve highly sensitive detection of low-concentration CO_2_. The sensing reaction is performed in water, and CO_2_ gas needs to be bubbled in the water of the cuvette to react with water to produce carbonic acid. By reacting with the tertiary amine group on the PDMAPMA (dimethylaminopropyl methacrylamide) polymer chain, the positively charged polymer chain generates electrostatic repulsion, which causes the hydrogel grating to swell, resulting in a photometric change and CO_2_ sensing. Subsequently, repeated purging of the hydrogel with an inert gas such as argon or nitrogen can remove the adsorbed CO_2_ from the surface, making the hydrogel grating reusable.

However, all of the above CO_2_ sensors require additional collection of the gas to be measured and injection into the reaction cell. This increases the monitoring steps and decreases the timeliness, and does not allow for real-time monitoring of the real air environment. The 3D electron-conductive hydrogel RGOH has a high specific surface area, which helps facilitate gas adsorption and enables direct sensing of gaseous CO_2_. Wu et al. [74] have developed a hydrogel material that can directly respond to CO_2_. They prepared three-dimensional chemically functionalized reduced graphite oxide hydrogels (FRGOH) using hydroquinone molecules by a simple one-step hydrothermal method to achieve high-performance sensing of CO_2_. The FRGOH device exhibits nearly twice as high response to CO_2_ (1.65% response to 1,000 ppm CO_2_) with a lower detection limit (20 ppm) compared to the unmodified RGOH sensor. When CO_2_ is adsorbed on the hydrogel surface, the highest occupied molecular orbital (HOMO) is above the Fermi energy level of the RGO. The charge is transferred from CO_2_ to RGO by mixing (hybridization) with RGO orbitals, leading to an increase in the charge (hole) concentration in the RGO and a decrease in the resistance of the hydrogel. As a result, hydrogel devices with higher initial resistance will have a larger response space and thus produce a higher response. However, this hydrogel CO_2_ sensor is not tolerant to high temperatures and an increase in temperature will reduce the response of the device.

### Explosives Sensors

Many regions and countries are plagued with public safety problems caused by explosives-based terrorist attacks. In addition to directly endangering the property security and life safety of the country and its citizens, explosives may also cause damage to human health through long-term or short-term exposure [238]. If the presence of explosives can be detected before an explosion occurs, tragedies such as human casualties and property damage can be minimized or even avoided. Typically, explosives are very sensitive to friction or impact, so it is critical to develop sensing devices that can detect explosives quickly, sensitively, accurately, and without contact. In addition, most explosives have extremely low vapor pressure at room temperature and adsorb to surfaces with high surface energy (such as metals), which are easily concealed and lack detectable characteristics [238–240]. Therefore, the detection limit of the sensor has higher requirements. Available sensing equipment for the detection of trace explosives such as ion mobility spectroscopy (IMS), mass spectrometry, gas chromatography (GC), GC coupled with MS (GC–MS), surface-enhanced Raman spectroscopy (SERS), nuclear quadrupole resonance (NQR) and energy dispersive X-ray diffraction (EDXRD), although with high selectivity, but bulky, expensive, not conducive to set in public places for the real-time monitoring of explosives [241–248]. The development of low-cost, portable, wearable, highly sensitive, and selective trace explosives sensors is indispensable for safeguarding people’s lives by helping them to stay away from hazardous areas with potential explosion risks.

Generally, changes in electrical parameters can be used as sensing signals for the target. Puttasakul et al. [249] prepared electrochemical gas sensors using PAM hydrogels for the detection of explosive substances. They first made PAM hydrogels and then cut the gels into 1 cm^2^ slices and immersed them in phosphate-buffered saline (PBS) containing 1:1 potassium ferricyanide (K_4_Fe(CN)_6_) and potassium ferricyanide(K_3_Fe(CN)_6_). Subsequently, sensing data were collected by linear scanning voltammetry (LSV) to monitor the explosive vapor signal by tracking the oxidation peak currents of the redox couples K_3_Fe(CN)_6_ and K_4_Fe(CN)_6_. The analysis revealed that the type and concentration of molecules adsorbed on the hydrogel surface can change the rate of electron transfer in Fe^2+^/Fe^3+^ redox. Different gas phase structures interacted differently with the acrylamide side chain, resulting in increased or decreased electron rates in the Fe^2+^/Fe^3+^ redox reaction. Each compound has a different rate of current change, and different samples can be identified by the current change. However, the hydrogel sensor has not undergone any functionalization, and it is easy to lose water and become dry during the actual detection process, and also cannot work properly at higher temperatures for a long time, which needs further improvement and optimization.

**Fig. 11:**
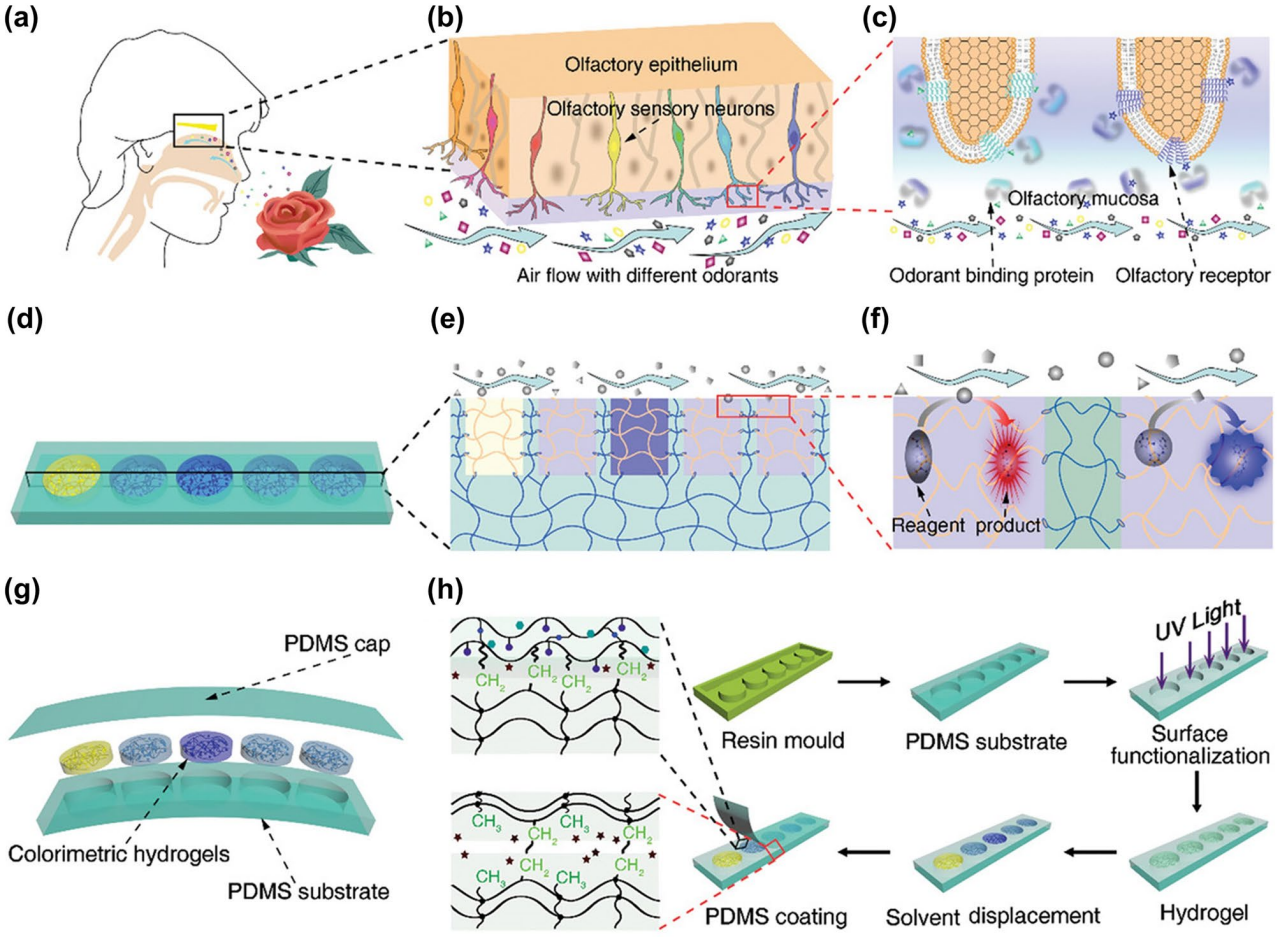
**a-c** Diagram of the human olfactory system for olfactory signal transmission. **d-f** Diagram of the sensing process of the colorimetric hydrogel sensor on the target substance. **g** Structure and **h** fabrication of colorimetric hydrogel sensor. Reproduced with permission [250]. Copyright 2020, Wiley–VCH

In addition to electrical parameters, adding colorimetric solutions to hydrogels to achieve the detection of target substances by color change is another common method. Imitating the human olfactory system, Wang et al. [250] have developed a multifunctional hydrogel detector that enables the detection of a wide range of explosive substances (Fig. 11a-c). They combined hydrogels with colorimetric reagents and synthesized hydrogels in situ after adding colorimetric reagents on the PDMS substrate, as shown in Fig. 11g-h. The hydrogel was firstly used to simulate the olfactory mucosa to adsorb and deliver the target microparticles. Then specific colorimetric reagents are used to simulate odor-binding proteins that bind to specific target microparticles (Fig. 11d-f). Finally, different specific colors are used as identification signals to discriminate different gases. The resulting hydrogel sensor was able to distinguish five different simple igniters, including hypochlorite, chlorate, perchlorate, urea and nitrate, at room temperature. The sensor is able to recognize concentrations as low as 39.4 pg with a response time of about 0.2 s. However, the response signal of this sensor is an optical signal. Different colors are used to mark the different simple explosives, and then the concentration of the target gas is determined by the shade of the color. The recognition process of the response signal is complicated and additional processing is required to obtain detailed gas information, such as the gas concentration.

## Hydrogel-based Humidity Sensors

A humidity sensor is a device that can detect the moisture content in the environment. It has applications in meteorology, hydrology, medicine, biology, agriculture, forestry, and production and storage of various products [261–266]. Conventional humidity sensors usually suffer from low mechanical deformability, low transparency, and time-consuming production processes. In contrast, flexible hydrogel-based humidity sensors have higher stretchability and transparency, making them more comfortable for the users. Thus, considerable progress has focused on the research of hydrogel-based humidity sensors in the fields of electronic skin, respiratory monitoring, and environmental humidity detection. In this section, recent development of hydrogel-based humidity sensors from the perspective of their application is reviewed, along with a brief introduction to their humidity-sensing mechanism. The performance parameters of the hydrogel-based humidity sensors mentioned in this section are summarized in Table 2.

**Table 2:** Sensing performance of hydrogel-based humidity sensors in this review

Material	Application	RH range (%)	t_res_/t_rec_	Response	Deformability	Temp	Refs
CS hydrogel	EHM	0–90	141/140 ms	Color change	\	RT	[267]
DSA-DMAEM- MBAA	EHM	0–100	10 s/20 min	Color change	\	RT	[271]
PHEMA	EHM	3–97	≤ 2.5 s/none	Color change	\	RT	[272]
PAM/Carr-EG-Gly	EHM	4–90	0.27/0.3 s	968%@90% RH	1225% strain	RT	[270]
starch/PAM	EM	35–97	\	20,313%@97% RH	50% strain	RT	[276]
CGGN	EM	20–90	\	97.1%@90% RH	> 440% strain	RT	[78]
PAM/Carr- LiBr	EM	11–98	201/41 s	454,414%@98% RH	100% strain	RT	[152]
PVA/CNF	EM	11–98	380/140 s	25,000%@98% RH	350% strain	RT	[277]
PAM/cassava gum- polyol -LiBr	EM	11–98	275.6/227.0 s	13,462.1% RH%^−1^	97% strain	RT	[206]
PAM/Eg	E-skin	13–85	3/94 s	–	400% strain	RT	[284]
ICOH	E-skin	45–85	–	0.35% RH%^−1^	> 400% strain	RT	[285]

t_res_/t_rec_: Response time/recovery time; Temp.: Operating temperature; RT: room-temperature; EHM: Environmental humidity monitoring; EM: Exhalation monitoring

### Humidity-Sensing Mechanism

**Fig. 12:**
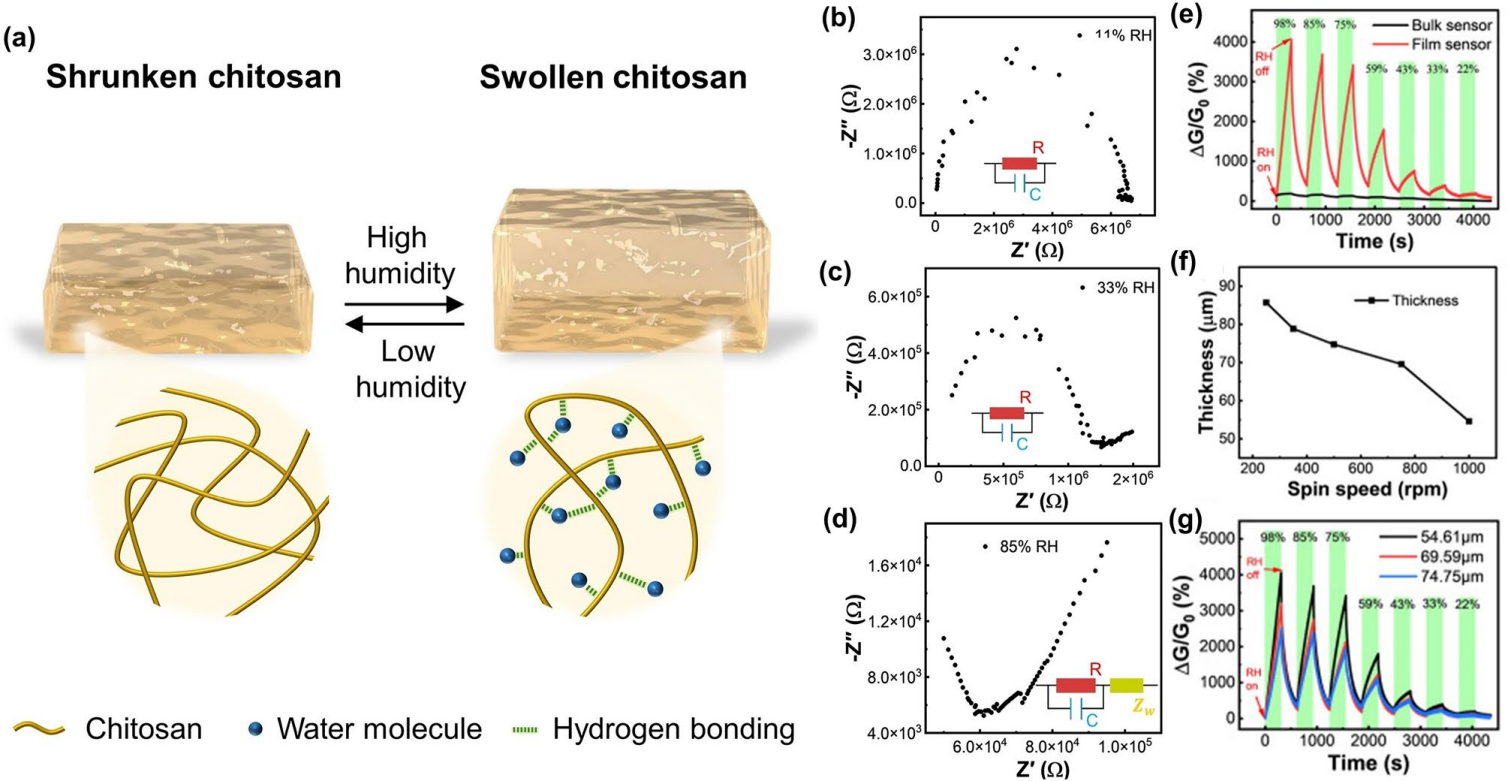
**a** Swelling/contraction of CS hydrogel under different humidity conditions. Reproduced with permission [267]. Copyright 2022, American Association for the Advancement of Science. **b–d** Complex EIS spectra of the humidity sensor under different RH environments. **e** Dynamic response curves of the thin-film and bulk hydrogels at different RH values. **f** Variations in the thickness of hydrogel with the spin-coating speed. **g** Dynamic response curves of the hydrogel films of different thicknesses at different RH values. Reproduced with permission [206]. Copyright 2022, Springer Nature

The humidity response of hydrogel cannot be separated from their swelling properties, which is pertain to the increases in gel volume after absorbing liquid. Taking the sandwich hydrogel humidity sensor prepared by Jung et al. as an example [267], the hydrogel can absorb water molecules of more than 1,000 wt% of its own dry weight under the action of hydrogen bonding, which rapidly expand as it become thicker. Figure 12a shows the increasing thickness of the hydrogel and gradually decreasing refractive index with increasing relative humidity of the external environment. Macroscopically, the color of the hydrogel resonator changes as the ambient humidity changes, which allow humidity detection.

Solubilization can affect the electrical conductivity of the hydrogel. As the ambient humidity increases, the hydrogel absorbs more water molecules, internal conductive channels are formed, the conductivity grows increases, and the electrical parameters vary, which allows the recognition of different humidity levels. Liang et al. [206] used electrochemical impedance spectroscopy (EIS) to detect the impedance changes of hydrogels at different humidity levels and confirmed their effects on the conductivity of hydrogels. As shown in Fig. 12b, the complex EIS spectrum becomes semicircular under a dry environment. The amount of water molecules adsorbed by the sensitive film is small, and ion-conducting channels are not formed. When the humidity increases to 33% RH, the complex EIS spectrum still exhibits a complete semicircle. However, the graded Warburg impedance is introduced, and short diagonal lines appear in the low-frequency region, as shown in Fig. 12c. Nevertheless, the changes of the Warburg impedance to resistance and conductivity of the hydrogel are minimal. When the humidity was continuously increased to 37–98% RH, the semicircular part of the complex EIS spectrum gradually disappeared, and the slash part was gradually elongated, as shown in Fig. 12d. In this humidity range, the Warburg impedance becomes the main factor of the EIS spectrum, and the hydrogel-sensitive film absorbs more water molecules, thereby forming a continuous hydrated layer inside, in which ions can directly diffuse and form conductive channels, and the conductivity increased with the humidity. Furthermore, the humidity response of the hydrogel was highly dependent on its thickness. Spin coating, combined with sol–gel method, was used to prepare thin-film hydrogel humidity sensors with thicknesses of a few microns and sensitivity of up to 13,462.1% RH%^−1^, which exceeds that of bulk hydrogels (Fig. 12e). The sensitivity of the hydrogel film to the humidity significantly increased as the thickness of the film was decreased owing to the increase in the specific surface area and initial resistance, as shown in Fig. 12f–g.

### Environmental Humidity Monitoring

Apart from various toxic and flammable gases, the measurement and control of RH are also important in environmental monitoring. For example, in museums and libraries, humidity monitoring and control facilitates the preservation of artifacts, paintings, sculptures or books, while in industrial applications such as semiconductor, textile and food production, humidity monitoring is also required [268, 269]. Moreover, the environmental humidity also has a great impact on human health. When RH is less than 45%, the dry air tends to evaporate a lot of water from the respiratory mucosa of the nose and lungs, making the throat dry and even congested nasal mucosa, triggering bronchitis, bronchial asthma and other respiratory diseases. Whereas when RH is greater than 80%, excessive humidity will affect the body’s thermoregulatory function, making the body’s water evaporation slower and more difficult to dissipate heat, resulting in chest tightness and shortness of breath and even inducing acute aggravation of cardiovascular and cerebrovascular diseases.

**Fig.13:**
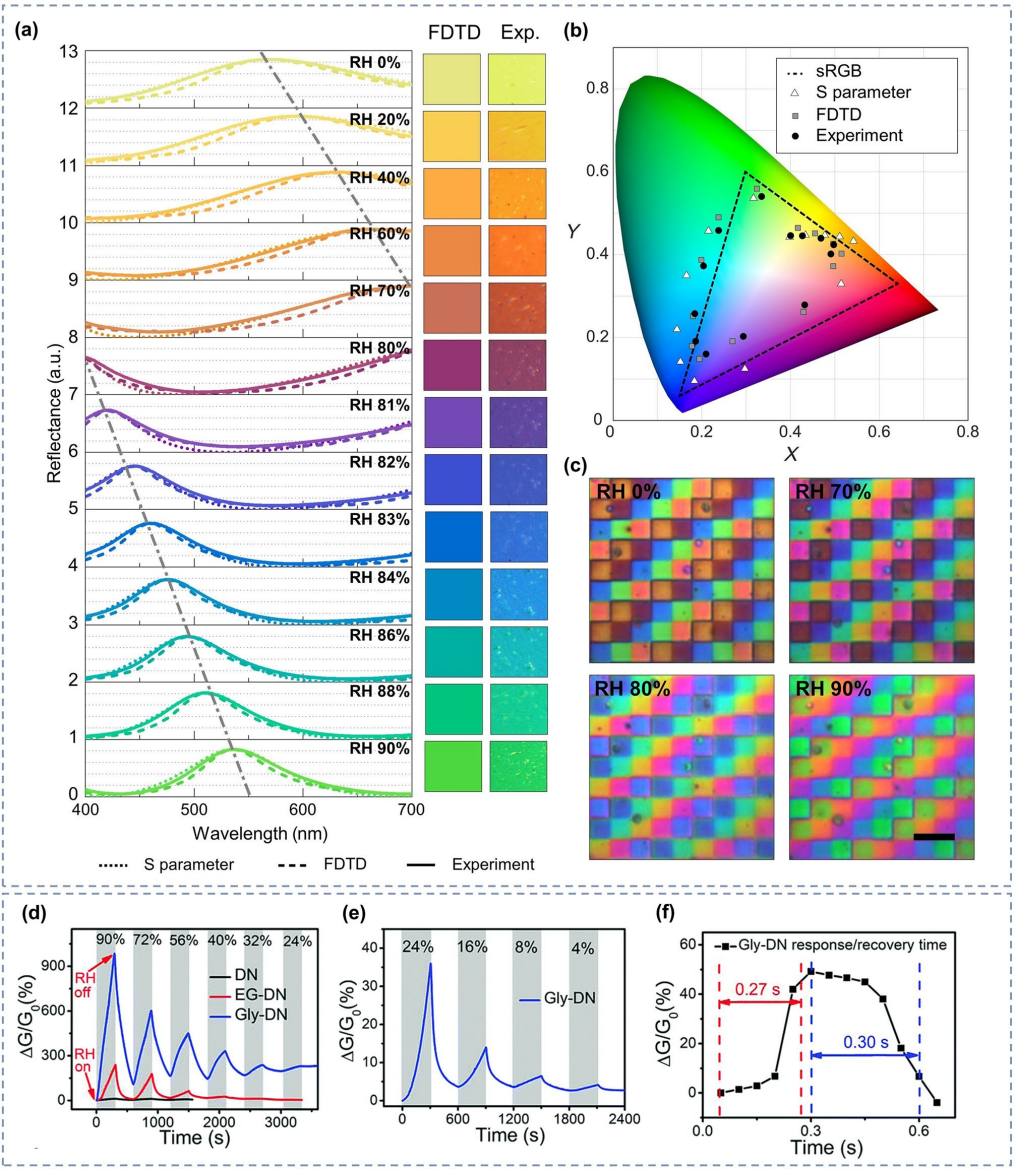
**a** Optical responses (reflection spectra) of hydrogel sensors in different RH values. **b** CIE 1931 color space of the XY values converted from the reflection spectra. **c** Hydrogel sensors for high-resolution displays: the color of the display changes with the ambient humidity. Reproduced with permission [267]. Copyright 2022, American Association for the Advancement of Science. **d** Responses of the DN hydrogel and EG/Gly-DN organohydrogels to different RH values. **e** Response of the Gly-DN organohydrogel to low RH values. f Response/recovery time of respiration monitoring. Reproduced with permission [270]. Copyright 2019, Royal Society of Chemistry

Because hydrogels have solvation properties and their volume expands with increasing ambient humidity, many studies have taken advantage of this property to design humidity sensors parameterized by optical signals. Jung et al. [267] prepared an ultra-fast panchromatic colorimetric hydrogel humidity sensor by using CS hydrogel as the middle sandwich and disordered metal NPs and reflective substrates as the two sides of the sandwich. The sensor uses the swelling property of hydrogel in different humidity atmospheres to sense the environmental humidity, and the sensor will show different colors in different RH as demonstrated in Fig. 13a. The color variation of a single sensor is very diverse and can cover 90% of the standard red, green, and blue (sRGB) gamut in CIE 1931 color space (Fig. 13b), making it also useful as a high-resolution display, as shown in Fig. 13c, where we can visually see the color display variation due to humidity. Tellis et al. [271] developed a fluorescence-based relative humidity measurement scheme using environmentally sensitive fluorophores embedded in hydrogels. They incorporated phenoxy sulfonic acid (DSA) fluorophores in two different hydrogel membranes (agarose and a copolymer of acrylamide and 2-(dimethylamino)ethyl methacrylate (DMAEM) crosslinked with MBAA). The expansion and contraction of the hydrogels at different RH changed the environmental polarity of the DSA, stimulating the shift of the emission wavelength and thus generating the response signal. In addition, hydrogel films of PHEMA on the order of several hundred nanometers were prepared by Buchberger et al. [272] using chemical vapor deposition. They used Flory–Huggins theory to describe the variation of the relative thickness of the hydrogels in relation to the RH in the environment. The thickness of the hydrogels increased significantly when they were in contact with humid air. Interferometric measurements of the hydrogel thickness variation were achieved using both laser and tube-band light sources. They determined the relevant interference constant x for PHEMA in combination with water over a wide range of humidity, thus enabling the application of this hydrogel sensor for humidity sensing in the range of 3 to 97% RH.

In addition to volume expansion, the electrical parameters of the hydrogel also vary with ambient humidity, and the electrical signal is more accurate and intuitive compared to observing color changes with the naked eye, as well as facilitating subsequent data processing. Wu et al. [270] prepared an electrochemical humidity sensor that can be directly sensitive to humidity. They used Eg/Gly modified PAM/Carr organohydrogels to prepare ion-conductive hydrogel-based humidity sensors with high stretchability (1.225% strain), self-healing properties and transparency. Because of the easy formation of hydrogen bonds and fine polymer chains between water molecules and a large number of hydrophilic groups, this organohydrogel humidity sensor exhibits high sensitivity and a wide humidity detection range (4–90% RH) with fast response (0.27 s) and recovery (0.3 s) rates, as shown in Fig. 13d-f. The presence of the highly hygroscopic Eg/Gly molecules, which form strong hydrogen bonds with water, effectively inhibits the evaporation of water, thus making the sensor free from the problems of easy water loss and instability of conventional hydrogels.

### Exhalation Monitoring

Water is one of the important components of exhaled breath. By monitoring the concentration of water vapor, the identification of respiratory characteristics can also be achieved to further predict sleep apnea, asthma, anemia and other chronic lung diseases for the early detection and treatment [273–275]. However, existing medical respiratory analysis equipment is expensive and requires specialized knowledge to operate, which is not conducive to real-time monitoring of patient breathing. Therefore, there is a demand to develop portable and wearable respiratory detection devices to monitor the humidity level in human exhaled breath in real time to achieve non-invasive early identification and tracking of diseases.

**Fig. 14:**
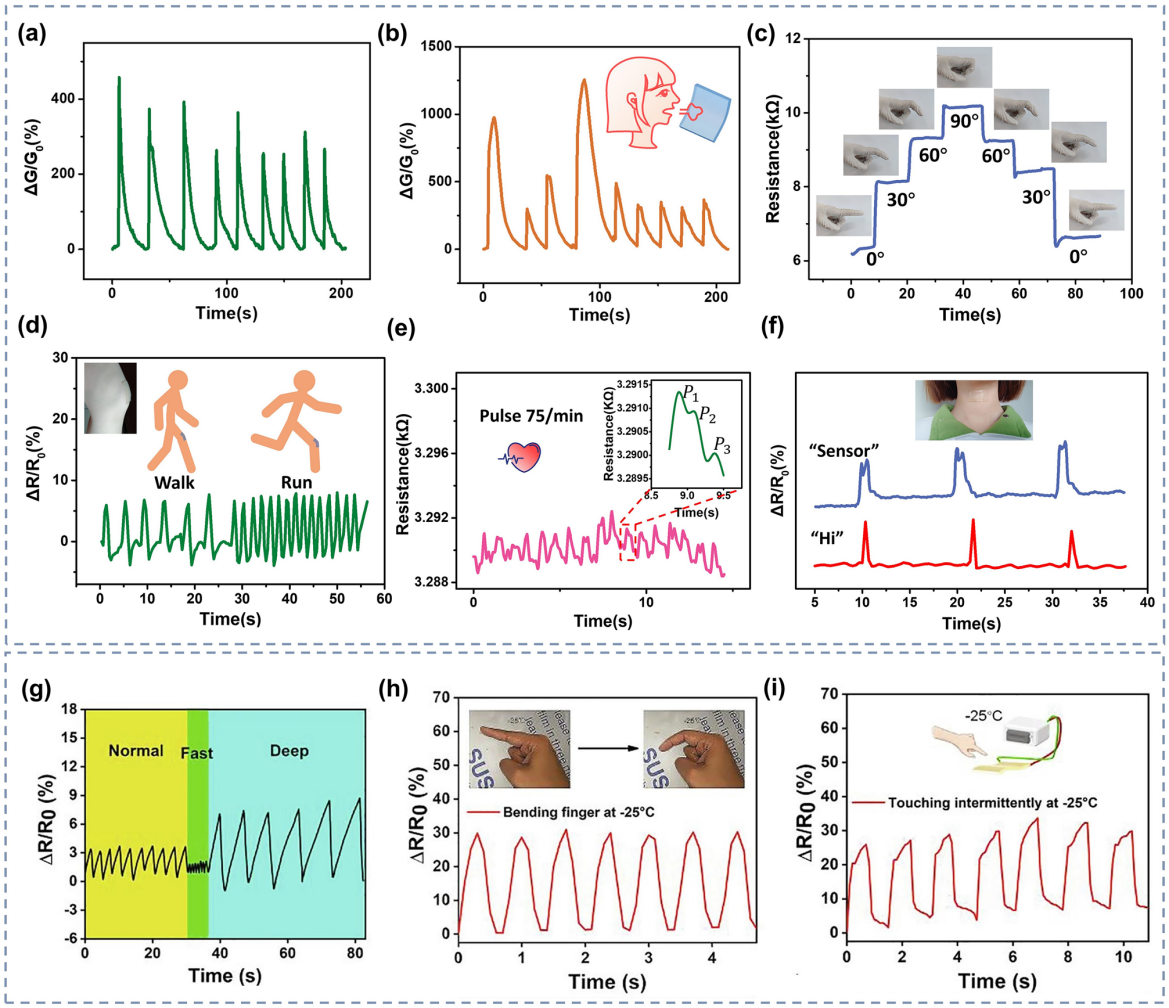
Response of the humidity sensor to **a** regular and **b** irregular breath. Response to **c** finger bending, **d** knee bending at different frequency, **e** pulse at the wrist and **f** vocal cord vibration. Reproduced with permission [276]. Copyright 2021, Elsevier. **g** CGCN sensors identify different patterns of breathing. **h** Bending finger and **i** touching intermittently at – 25 °C. Reproduced with permission [78]. Copyright 2021, Elsevier

Since most functionalized hydrogels have multi-sensitive properties and can be sensitive to temperature, pressure and humidity at the same time, many functionalized hydrogel materials are often used to prepare multi-functional sensors. Zeng et al. [276] used a simple one-step method to develop a transparent and highly flexible starch/PAM DN hydrogel humidity sensor based on natural renewable starch. The hydrogel-based sensor is highly flexible and can withstand 80% compressive strain and 135% tensile strain. At the same time, it is humidity sensitive over a relative humidity range of 35 to 97%. After 100 times of 50% compressive strain, the hydrogel-based sensor still works properly, demonstrating its potential for stable operation in real-world applications. The authors also did real-time monitoring tests on human respiration, and the sensor performed good recognition of exhalations of different lengths and intensities, as shown in Fig. 14a-b, implying its potential application in respiratory monitoring. Moreover, the hydrogel-based sensor has a good response to strain and can be used for finger bending, standing/running posture recognition, heartbeat frequency detection and vocal cord vibration recognition (Fig. 14c-f). Using CS, gelatin, and Gly as raw materials, Gao et al. [78] prepared an organohydrogel film (CGGN) with only 0.1 mm thickness for humidity sensing. The sensor is capable of detecting relative humidity from 20 to 90%. Because of the high sensitivity to humidity, the sensor is capable of fast, stable, continuous, and reproducible monitoring of human respiration, as Fig. 14g showed. For a single breath, the response and recovery times of the sensor are 0.41 and 0.3 s, respectively. Meanwhile, the sensors are equally sensitive to temperature and pressure, and the binary solvent of water and Gly makes the hydrogel sensor resistant to drying and freezing and can even work stably at − 25 °C, as depicted in Fig. 14h-i. The ultra-thin, stretchable and highly transparent properties allow the CGGN sensor to fit perfectly on the human skin surface without affecting the aesthetics.

**Fig. 15:**
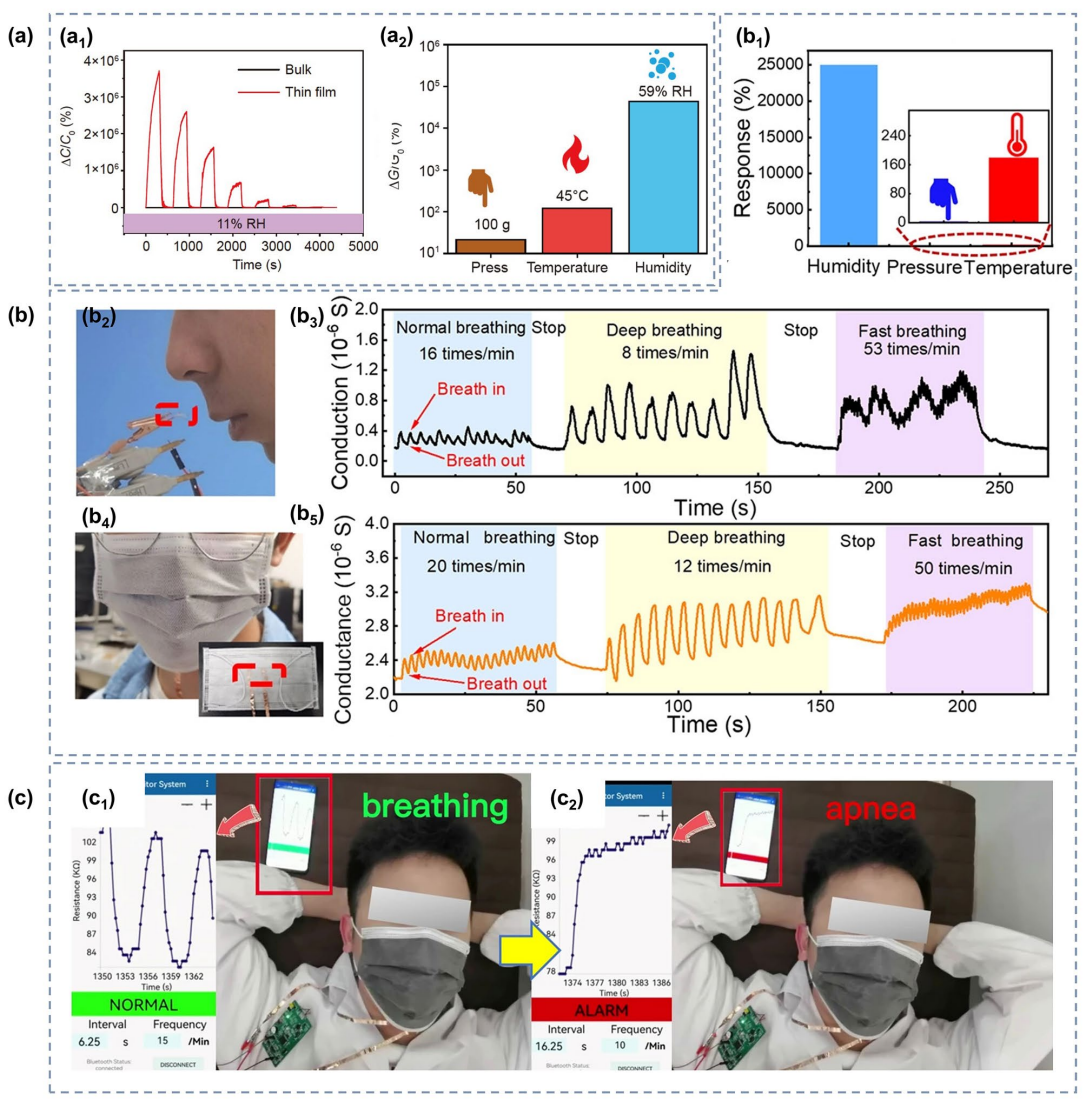
**a**_**1**_ Comparison of humidity response of film and block hydrogels. **a**_**2**_ Comparison of the response of different sensitive sources. Reproduced with permission [152]. Copyright 2022, Springer Nature. **b**_**1**_ Comparison of the response of different sensitive sources. **b**_**2**_ Schematic diagram of the handheld breath test. **b**_**3**_ Real-time response curve of handheld breath test in different breathing modes. **b**_**4**_ Schematic diagram of the mask breathing test. **b**_**5**_ Real-time response curve of the mask breathing test in different breathing modes. Reproduced with permission [277]. Copyright 2023, Wiley‐VCH. **c**_**1**_**-c**_**2**_ Monitoring of “normal breathing” and “apnea” with smart masks. Reproduced with permission [206]. Copyright 2022, Springer Nature

However, the identification of multiple sensitive sources is still a problem that cannot be ignored, and the multifunctional sensor cannot be truly applied in practice if the response signal changes without being able to distinguish the source. It is better to enhance the selectivity of hydrogel-based humidity sensing first, and then realize multifunctional sensing by other designs subsequently. Wu et al. [152] fabricated PAM/Carr hydrogel films of different thicknesses by tuning the suspension rate and integrated them on plasma-treated PDMS substrates to prepare intrinsically stretchable, ion-conductive, highly transparent and high-performance humidity sensors. To further enhance its resistance to drying and freezing, LiBr solution was introduced into the hydrogel. The thin film hydrogel shows higher humidity response than the bulk one, and it has a sensitivity up to 78,785.5% RH%^−1^, as shown in Fig. 15a_1_. With the benefit of ultra-high humidity response, this hydrogel sensor exhibits relatively good humidity selectivity, but still cannot exclude the interference of temperature and strain (Fig. 15a_2_).

Ding et al. [277] developed a humidity-sensitive ionic skin composed of PVA/CNF organohydrogel film with controllable thickness prepared by a simple combined one-pot and spin-coating method. The sensor features excellent stretchability, self-healing ability, high transparency and high response to humidity (25,000 to 98% RH). It can withstand different mechanical deformations and still maintain the sensing function. Furthermore, it has the ability to self-repair, and can self-heal after cutting, without affecting its sensitivity. More importantly, the sensor is extremely selective for humidity, as the Fig. 15b_1_ illustrates. The authors also tested the suitability of the sensor application for breath detection under different conditions. The humidity sensor can monitor the breathing well whether it is hand-held or worn with a mask, demonstrating its possibility in practical application, as shown in Fig. 15b_2_-b_5_. Liang et al. [206] prepared a transparent DN hydrogel with good mechanical deformability using PAM and cassava gum as raw materials, and introduced polyol and LiBr to further enhance its stability and reliability. The hydrogel sensor has a high sensitivity of 13,462.1% RH%^−1^ to humidity and can still operate in the stretched state. By integrating this hydrogel sensor, self-designed circuitry, and the mask, the authors also fabricated a mask that allows wireless breathing monitoring, as shown in Fig. 15c_1_-c_2_. The mask is able to recognize breathing patterns, demonstrating its potential application in sleep apnea disorders monitoring.

### E-skin

Human skin is a complex and powerful organ in the human body. It separates us from our surroundings and allows us to have the ability to sense temperature, humidity, and various pressures, shapes, and patterns. While e-skin is a smart skin that mimics human skin by integrating various sensors. It has a wide range of applications in the fields of robotics, digital health, fashion, and the Internet of Things. In addition, many electronic skins have a sensitivity far higher than that of human skin. They could be attached to the surface of the human body to measure various body parameters or environmental parameters, expanding people’s knowledge of themselves and their environment, acting as a human second skin [278–283].

Most hydrogel-based electronic skins, in order to more realistically mimic human skin, are generally multi-sensitive, capable of sensing humidity, temperature, and deformation simultaneously. Inspired by human skin, Ying et al. [284] designed a new hydrogel ionic electronic device to develop artificial ionic skin (AIskin) with unprecedented properties. They combined physically crosslinked agarose and covalently crosslinked PAM and added Eg to form a bilayer DN hydrogel with opposite charges. The e-skin simulates a diode based on controlled ion mobility, similar to the transmembrane ion transport of neuronal sensors in human skin. It has high toughness, good stretchability, high environmental stability and high transparency. AIskin can withstand over 400% strain and is sensitive to both strain and humidity. The authors demonstrated experimentally that AIskin could convert strain and humidity stimuli into resistance, capacitance, open circuit voltage and short circuit current signals, the latter two of which can be generated spontaneously. However, this AIskin senses humidity in the range of 13 to 65% RH, when the RH is greater than 65%, the response will tend to saturate. To further extend the range of humidity monitoring, by imitating the human skin system, Pan et al. [285] developed a nanocomposite enhanced ion-conductive organic hydrogel (ICOH) that can be used for smart electronic skin applications. The hydrogel is sensitive to humidity in the range of 45 to 85% and can be laminated to the skin to monitor skin humidity, as well as for environmental humidity monitoring. In addition, it has high transparency of 93.8%, high stretchability of 450%, frost resistance that does not freeze even at – 63.28 °C, dryness resistance, electrical conductivity and thermal self-healing ability, and can adapt to complex and diverse usage environments.

**Fig. 16:**
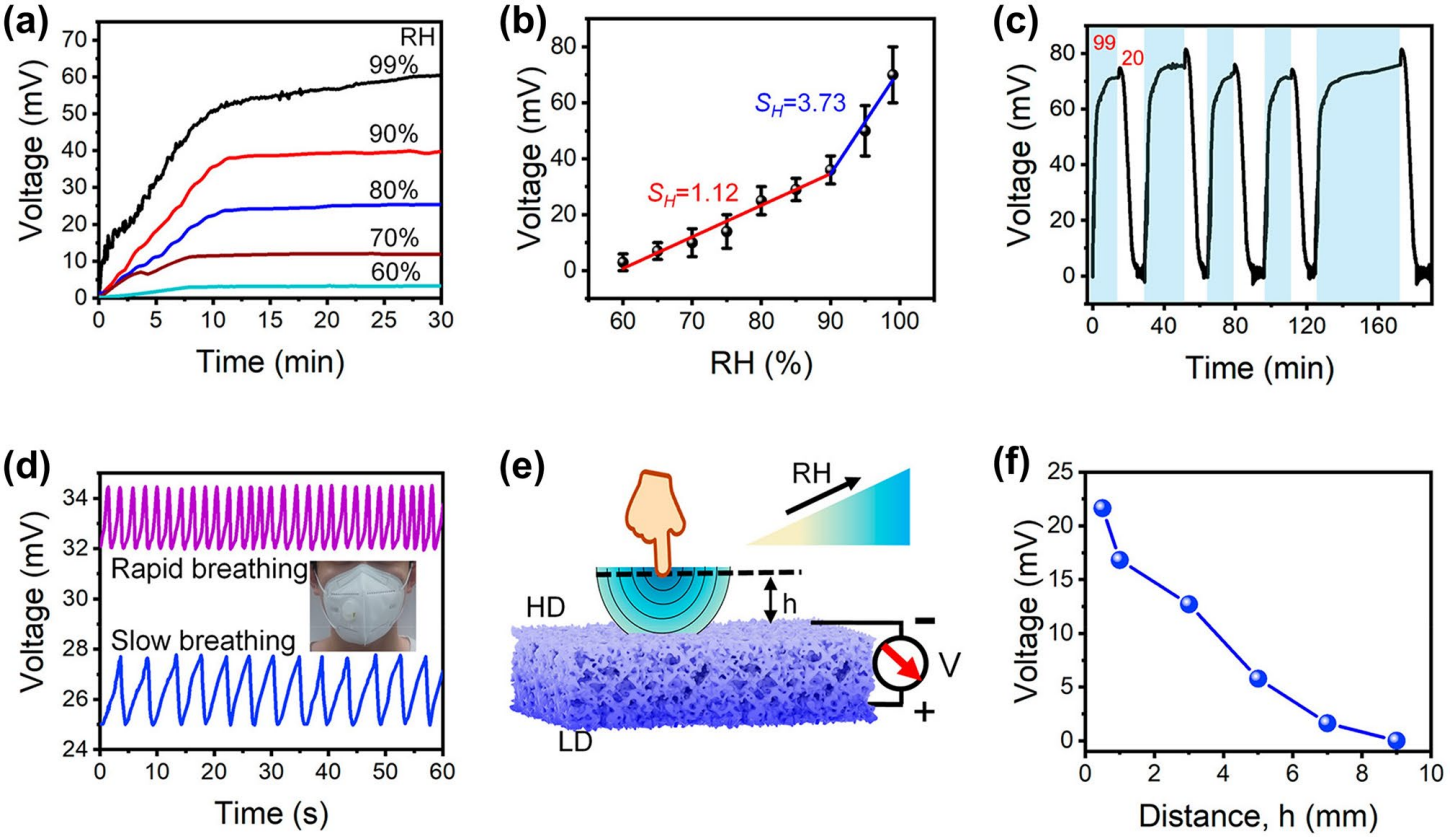
**a** Output voltage of I-skin at different RH. **b** Humidity sensitivity of I-skin. **c** Output voltage of I-skin at 99% RH and 20% RH alternately. **d** Output voltage variation curve of I-skin under different breathing modes. **e** Diagram of finger distance perception by I-skin through humidity change. **f** Variation of output voltage of I-skin at different finger distances. Reproduced with permission [79]. Copyright 2022, American Chemical Society

However, the above e-skin requires additional power devices to provide energy, which limits the application of e-skin and is not in line with its skin characteristics. Therefore, the study of self-powered electronic skin is also of great interest. The basis of self-powering is to create a potential difference between the electrode ends of the hydrogel. One approach is to use electrode materials with differential work function as the sensing electrode and counter electrode of the sensor, and another is to make the hydrogel itself with potential difference. Xia et al. [79] developed a self-driven human-like ionic skin (I-skin) based on gradient polyelectrolyte membranes (GPMs). The GPMs were prepared under a hydrogel-assisted reactive diffusion method with polymer network species presenting charged groups in a concentration gradient distribution. In the case of dehydration/drying, GPMs have moisture-sensitive self-induced potentials, which enable a response to humidity. When the device is exposed to a humid environment, the polar groups gradually hydrate and the counter ions spontaneously diffuse, leading to a gradual increase in the self-induced voltage of the I-skin, as shown in Fig. 16a. At lower humidity below 90% RH, the humidity sensitivity of I-skin is 1.12 mV RH%^−1^, which increases to 3.73 mV RH%^−1^ when the RH is higher than 90%, as shown in Fig. 15b. The authors integrated the I-skin into a commercial respirator and verified its respiratory monitoring capability (Fig. 15d). In addition, this I-skin could also sense the distance of the human body based on human skin humidity, as shown in Fig. 16e-f.

## Conclusion and Perspective

In this review, we briefly described the properties and performance optimization methods of hydrogels. From the point of view of gas and humidity sensing, different types of gas and humidity sensors for personal health and safety monitoring, along with their sensing mechanisms were introduced. The intrinsic conductive, flexible, stretchable, self-healing, self-adhesive, and biocompatible properties of functionalized hydrogels were demonstrated, which are suitable for wearable sensor applications with higher comfort than that of traditional rigid sensors. Moreover, hydrogel-based sensors that can detect various gases and humidity values at room temperature, which greatly reduce the power consumption and risks associated with high-temperature operation, are discussed. Currently, studies related to hydrogel-based gas sensors for the detection of NO_2_, NH_3_, O_2_, CO_2_, and explosive gases have been conducted. The gas-sensitive response usually occurs at the interface between the hydrogel and electrode, where the target gas undergoes a redox reaction, resulting in a change in the electrical properties and response signal. In addition, the ambient humidity affects the aerodynamic response of the hydrogels, which are more likely to adsorb more target gas under high-humidity conditions, thereby producing a larger response signal. With the long history of hydrogel-based humidity sensors, flexible hydrogel humidity sensors have been widely employed for environmental monitoring, healthcare, and electronic skin. The humidity-sensing process cannot be accomplished without the swelling properties of the hydrogels, which increases the water content inside the hydrogel with the humidity of the environment. Subsequently, changes in the hydrogel volume can affect the optical signal, whereas conductive channels formed within the hydrogel can affect the electrical signal; both of which can be used to generate a humidity-sensitive response signal. The hydrogel thickness also influences their humidity response. In particular, thin-film hydrogels have a higher response than that of their bulk alternatives. The response increases with decreasing thickness of the film.

However, the research on hydrogel-based vapor sensors is still in its infancy. Although several studies have presented a preliminary idea, various problems need to be addressed to realize the actual applications of hydrogel-based vapor sensors.

First, from the existing gas sensor-related research, most of the development on hydrogel-based gas sensors is focused on NO_2_, NH_3_, and O_2_, which should be expanded to achieve a perfect gas detection system. In addition, further research is needed to explore the possibility of hydrogel applications in sensing other gases. In view of the available gas-sensitive mechanisms, the modification of electrode materials and hydrogel functionalization to achieve suitable sensitivity to more gases are important topics in the field of hydrogel-based gas sensors. For example, considering the research experience on metal–oxide semiconductor gas sensors, specific modifications can be made to the electrode materials to achieve selective response to other target gases.

Second, current research on hydrogel-based gas and humidity sensors mainly focuses on the functionalization of the materials. Studies on gas- and humidity-sensitive mechanisms remain limited. Moreover, no comparatively unified conclusion on the gas- and humidity-sensing mechanism of hydrogels is yet to be reported. Electrode properties and ambient humidity affect the response of hydrogels. However, the influence of other factors, such as the crosslinking method, crosslinking density, and conductivity, on the gas-sensing performance of hydrogels has not been studied. In the future, the investigation of their role on the sensing mechanisms should be determined to improve the design and preparation of different hydrogel gas/humidity sensors. For example, the gas- and humidity-sensing behavior of hydrogels can be further investigated by in situ characterization methods, such as Raman spectroscopy, infrared spectroscopy, and EIS.

Third, the multiple sensitivities of hydrogels are both an advantage and disadvantage. On the one hand, it endows hydrogels with the possibility of multifunctional sensing. On the other hand, the response cannot accurately identify the sensitive source. Thus, the changes in the electrical or other properties caused by pressure, tension, temperature, humidity, and various gas stimuli may not be distinguished. Multiple sensitivities can only be meaningful in practical applications if the response of a sensitive source can be characterized. Therefore, the development and design of multifunctional hydrogel-sensitive materials that can recognize and distinguish different stimuli should be analyzed to realize the multisensitivity of hydrogel and its applications. For example, the sensitive signal loops of different sensitive sources can be varied by structure or circuit design to distinguish different sensitive sources. In addition, algorithms can be designed to analyze large amounts of response data to determine the data patterns of different sensitive sources.

Fourth, the stability of hydrogels is greatly affected by their environment. Although studies have been conducted on methods for improving the durability and stability of hydrogels, the water content in hydrogels gradually decreases with time, and their storage and working cycles remain short, compared to traditional dry gas-sensing materials. This attribute is detrimental to the development of hydrogel-based wearable sensors. In the future, to achieve the practical application of hydrogel-based sensors, increased research should be conducted on exploring the improved durability and stability of hydrogel sensors to benchmark the existing available wearable products.

To realize wearable applications, wireless circuits and sensor chips need to be integrated to form smart, miniature, integrated, flexible, and wearable devices. Most of existing research only focuses on the sensing material itself, and integrated wearable devices have rarely been reported. However, existing individual hydrogel-based vapor sensors are at the macroscopically visible level, which is not favorable for the miniaturization and large-scale integration of sensors. Moreover, the preparation process of hydrogel gas/humidity sensors lacks a unified set of standards, which make it difficult to exclude the effects of different preparation processes on the gas- and humidity-sensing properties. These problems have hindered the commercial applications of hydrogel-based gas/humidity sensors. Therefore, to promote the development of hydrogel-based vapor sensors, the size reduction, standardized preparation, and design of hydrogels for easy integration into reliable sensing systems are important topics for future exploration. For instance, standardized mass production of hydrogel sensors can be achieved by screen printing or introducing a planar process based on the manufacturing of semiconductor devices.
